# Activation of intestinal endogenous retroviruses by alcohol exacerbates liver disease

**DOI:** 10.1172/JCI188541

**Published:** 2025-05-13

**Authors:** Noemí Cabré, Marcos F. Fondevila, Wenchao Wei, Tomoo Yamazaki, Fernanda Raya Tonetti, Alvaro Eguileor, Ricard Garcia-Carbonell, Abraham S. Meijnikman, Yukiko Miyamoto, Susan Mayo, Yanhan Wang, Xinlian Zhang, Thorsten Trimbuch, Seija Lehnardt, Lars Eckmann, Derrick E. Fouts, Cristina Llorente, Hidekazu Tsukamoto, Peter Stärkel, Bernd Schnabl

**Affiliations:** 1 Department of Medicine, University of California, San Diego, La Jolla, California, USA.; 2 Department of Medicine, Division of Gastroenterology and Hepatology, Shinshu University School of Medicine, Matsumoto, Japan.; 3 Department of Molecular and Cellular Biology, The Scripps Research Institute, La Jolla, California, USA.; 4 Division of Biostatistics and Bioinformatics, Herbert Wertheim School of Public Health and Human Longevity Science, University of California, San Diego, La Jolla, California, USA.; 5 Institute of Neurophysiology, Charité Viral Core Facility, and; 6 Institute of Cell Biology and Neurobiology, Charité–Universitätsmedizin Berlin, corporate member of Freie Universität Berlin, Humboldt-Universität zu Berlin, and Berlin Institute of Health, Berlin, Germany.; 7 J. Craig Venter Institute, Rockville, Maryland, USA.; 8 Department of Pathology, Keck School of Medicine of the University of Southern California, Los Angeles, California, USA.; 9 Department of Veterans Affairs Greater Los Angeles Healthcare System, Los Angeles, California, USA.; 10 Department of Hepatology and Gastroenterology, St. Luc University Hospital, Catholic University of Louvain, Brussels, Belgium.; 11 Department of Medicine, VA San Diego Healthcare System, San Diego, California, USA.

**Keywords:** Gastroenterology, Hepatology, Microbiome, Molecular biology

## Abstract

Alcohol-associated liver disease represents a significant global health challenge, with gut microbial dysbiosis and bacterial translocation playing a critical role in its pathogenesis. Patients with alcohol-associated hepatitis had increased fecal abundance of mammalian viruses, including retroviruses. This study investigated the role of endogenous retroviruses (ERVs) in the development of alcohol-associated liver disease. Transcriptomic analysis of duodenal and liver biopsies revealed increased expression of several human ERVs, including *HERV-K* and *HERV-H*, in patients with alcohol-associated liver disease compared with individuals acting as controls. Chronic-binge ethanol feeding markedly induced ERV abundance in intestinal epithelial cells but not the livers of mice. Ethanol increased ERV expression and activated the Z-DNA binding protein 1 (Zbp1)–mixed lineage kinase domain-like pseudokinase (Mlkl) signaling pathways to induce necroptosis in intestinal epithelial cells. Antiretroviral treatment reduced ethanol-induced intestinal ERV expression, stabilized the gut barrier, and decreased liver disease in microbiota-humanized mice. Furthermore, mice with an intestine-specific deletion of *Zbp1* were protected against bacterial translocation and ethanol-induced steatohepatitis. These findings indicate that ethanol exploits this pathway by inducing ERVs and promoting innate immune responses, which results in the death of intestinal epithelial cells, gut barrier dysfunction, and liver disease. Targeting the ERV/Zbp1 pathway may offer new therapies for patients with alcohol-associated liver disease.

## Abstract 2

Endogenous retroviruses (ERVs) play a key role in alcohol-associated liver disease by disrupting the gut barrier; blocking the ERV-Zbp1 pathway restores gut health and reduces disease severity.

## Graphical abstract

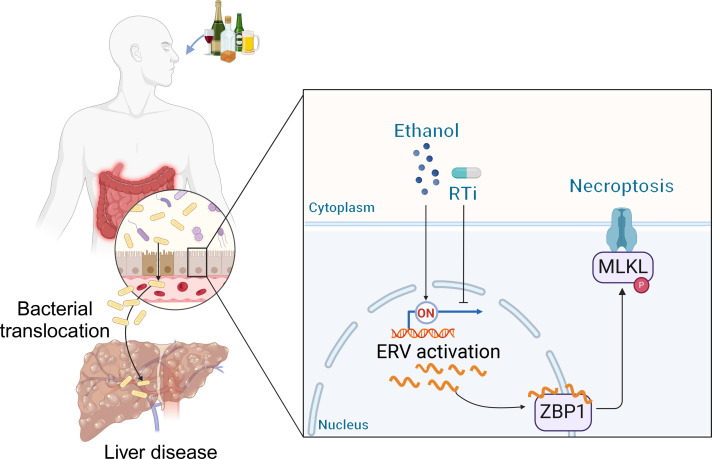

## Introduction

Alcohol-associated liver disease poses a significant global public health challenge, arising from prolonged and harmful alcohol consumption (1, 2). Chronic harmful alcohol consumption results in hepatic steatosis, which can progress to hepatitis, fibrosis, and cirrhosis. The gut-liver axis plays an important role in the pathogenesis of alcohol-associated liver disease (3). Alcohol consumption is associated with intestinal dysbiosis and gut barrier dysfunction. Microbial-associated molecular patterns can translocate from the gut to the liver, thereby contributing to liver inflammation and disease progression (4). Viable bacteria can also cross the gut barrier by mechanisms that are incompletely understood and contribute to alcohol-associated liver disease (5).

Our previous study revealed an increased abundance of mammalian viruses, including retroviruses, in the fecal virome of patients with alcohol-associated hepatitis (6). Despite this discovery, the specific role of the gut virome, particularly endogenous retroviruses (ERVs), in the development of alcohol-associated liver disease remains unknown. ERVs are ancient remnants of retroviral infections integrated into vertebrate genomes, including humans (7). Many human ERVs (HERVs), such as HERV-H and HERV-P (both gammaretrovirus-like), integrated into the genome over 30 million years ago, while some HERVs, such as HERV-K (betaretrovirus-like), entered the genome more recently (8–10). ERV genomes typically contain 3 main genes — *gag* (coding for the viral capsid), *pol* (encoding reverse transcriptase and integrase), and *env* (responsible for envelope proteins) — along with flanking long terminal repeats, though in most cases these sequences are nonfunctional, and no intact viral particles are produced (7). Originating from retroviruses infecting germ cells, ERVs are mostly located in the heterochromatin, have undergone silencing through DNA methylation mechanisms, and have lost their coding capacity (11). Recently, ERVs have been discovered as regulatory RNAs contributing to cellular homeostasis in a healthy state (12). Under certain circumstances, ERVs are transcribed and form double-stranded RNA (13). ERVs are sensed by the innate immune system and can contribute to disease, such as cancer, infection, or autoimmune disease (14–16). For example, high-fat diet–primed keratinocytes increase ERV expression and promote skin inflammation in response to commensal colonization, which is alleviated by antiretroviral therapy (16).

Z-DNA binding protein 1 (Zbp1) is a sensor for aberrant nucleic acids — such as noncoding RNA, misfolded RNA/DNA structures, or viral nucleic acids, including those derived from viral infections such as influenza A virus and cytomegalovirus (17). Zbp1 detects Z-RNA, a left-handed helical form of RNA. This structure can emerge under conditions of physiological stress or during viral infections. During influenza A virus infection, Zbp1 binds to viral Z-RNA, initiates receptor-interacting serine/threonine-protein kinase 3–mediated (Ripk3)-mediated activation, and triggers mixed lineage kinase domain-like pseudokinase (Mlkl)–dependent necroptosis (18). Necroptosis is therefore viewed as a part of the innate host defense to eliminate virus-infected cells (18, 19). Similarly, double-stranded RNA derived from ERVs triggers Zbp1 activation and activates Ripk3-dependent necroptosis (20). Patients with inflammatory bowel disease and reactivation of HERVs due to a dysfunctional upstream repressor SETDB1, experience activation of ZBP1 and the necroptosis pathway in intestinal stem cells, which leads to epithelial barrier damage and inflammation in the intestine (21).

In this study, we examined the role of ERVs and the involvement of Zbp1 in mediating the effect of ERVs on the progression of alcohol-associated liver disease.

## Results

### Patients with alcohol use disorder and liver disease have increased HERV expression in the duodenum and liver.

**Figure 1:**
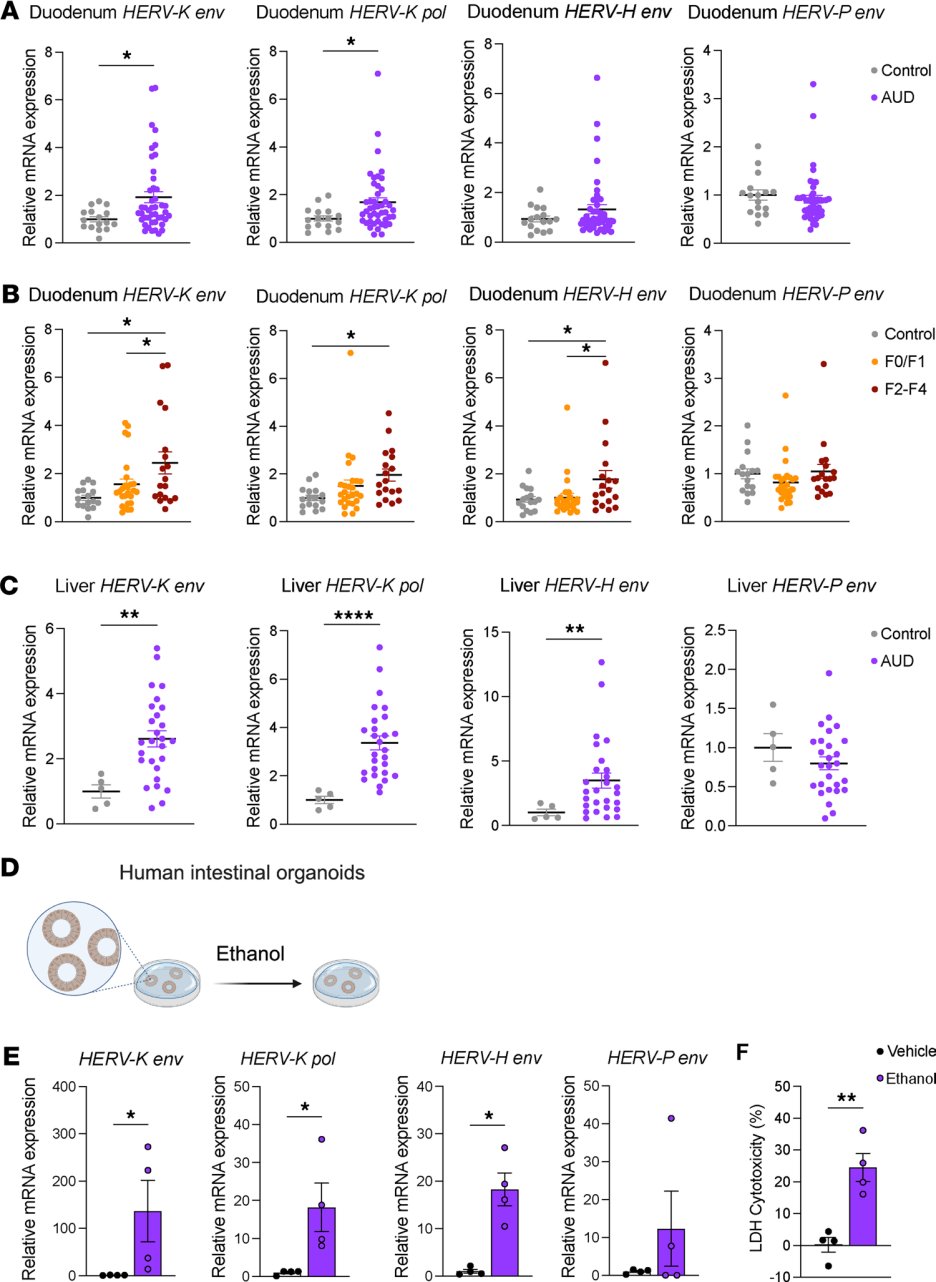
Upregulation of human endogenous retroviruses in duodenal biopsies of patients with alcohol-associated liver disease. (**A**) Duodenal biopsies were obtained from individuals without alcohol use disorder or alcohol-associated liver disease (controls; *n* = 16) and patients with alcohol use disorder and alcohol-associated liver disease (*n* = 44), and qPCR was performed to measure mRNA expression of human endogenous retroviruses (HERVs). (**B**) Duodenal expression levels of HERVs in patients with advanced fibrosis (F2–F4) compared with individuals acting as controls and patients with no or mild liver disease (F0–F1). (**C**) Liver biopsies were obtained from individuals without alcohol use disorder or alcohol-associated liver disease (controls, *n* = 5) and patients with alcohol use disorder and alcohol-associated liver disease (*n* = 27), and qPCR was performed to measure mRNA expression of HERVs. (**D**) Schematic representation of the experiment. (**D**–**F**) Human intestinal organoids were treated with ethanol (50 mM) for 24 hours to assess HERV expression (**E**) and to measure cytotoxicity using the lactate dehydrogenase (LDH) assay of human organoids (**F**). (**E** and **F**) Results were generated from 2 technical replicates. *P* values among groups were determined by Mann-Whitney U test (**A**, **C**, **E**, and **F**) or 1-way ANOVA with 2-stage step-up method of Benjamini, Krieger and Yekutieli test (**B**). Results are expressed as mean ± SEM. *P < 0.05, **P < 0.01, ****P < 0.0001.

To determine whether patients with alcohol use disorder and liver disease have increased HERV expression, we analyzed duodenal biopsies from individuals without alcohol use disorder or alcohol-associated liver disease (individuals acting as controls, *n* = 16) and patients with alcohol use disorder (*n* = 44) (Supplemental Table 1; supplemental material available online with this article; https://doi.org/10.1172/JCI188541DS1). Compared with individuals acting as controls, patients with alcohol use disorder showed a significant upregulation of *HERV-K env* and *HERV-K pol* genes (Figure 1A). Patients with progressive liver disease (steatohepatitis or steatofibrosis) had significantly higher expression of *HERV-K env* compared with those with nonprogressive disease (simple steatosis) or individuals acting as controls (Supplemental Figure 1A). Furthermore, patients with advanced liver fibrosis (F2–F4) had increased *HERV-K env*, *HERV-K pol*, and *HERV-H env* expression in the duodenum as compared with individuals acting as controls and patients with no or mild liver disease (Figure 1B and Supplemental Figure 1B). We also measured HERV expression in liver biopsies from individuals acting as controls (*n* = 5) and patients with alcohol use disorder (*n* = 27) (Supplemental Table 2). Patients with alcohol use disorder showed significant hepatic upregulation of *HERV-K env*, *HERV-K pol* and *HERV-H env* genes (Figure 1C).

To investigate the potential role of ethanol in modulating HERV expression, human intestinal organoids from 4 independent donors were incubated with ethanol (50 mM) (Figure 1D). Exposure to ethanol for 24 hours resulted in upregulation of *HERV-K env*, *HERV-K pol,* and *HERV-H env* expression (Figure 1E) and increased cytotoxicity, as determined by lactate dehydrogenase (LDH) release compared with vehicle-treated organoids (Figure 1F). These findings indicate that ethanol alone can directly induce HERV expression in intestinal epithelial cells (IECs).

### Ethanol-induced ERV expression activates Zbp1 and induces Mlkl-mediated necroptosis in IECs.

**Figure 2:**
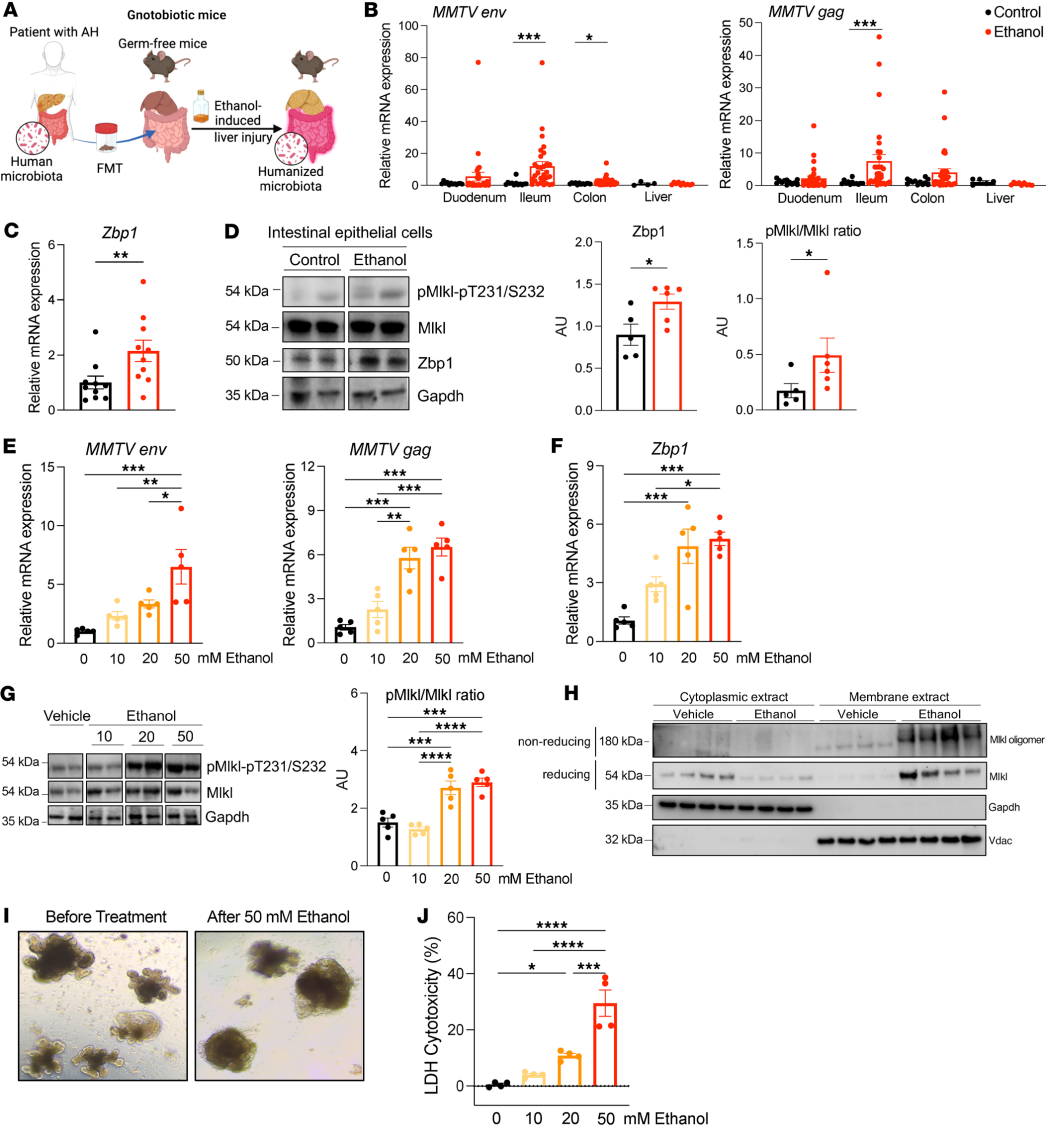
Ethanol activates Zbp1 and induces Mlkl-mediated necroptosis in intestinal epithelial cells. (**A**) Germ-free C57BL/6 mice were colonized with fecal microbiota from patients with alcohol-associated hepatitis (AH) and fed oral isocaloric (control) or chronic-binge ethanol diets. (**B**) Intestinal and hepatic levels of mouse mammary tumor virus (*MMTV*) *env* and *gag* mRNA. (**C**) Level of *Zbp1* mRNA in the ileum. (**D**) Intestinal epithelial cells were isolated from ethanol-fed mice, and immunoblots were performed for phospho-Mlkl, Mlkl, Zbp1, and Gapdh; protein amounts of Zbp1 relative to Gapdh and protein amounts of phospho-Mlkl relative to Mlkl are shown (*n* = 5 control, *n* = 6 ethanol). (**E–J**) Mouse intestinal organoids were incubated with ethanol (0, 10, 20, and 50 mM) for 24 hours. (**E**) Expression levels of *MMTV env* and *gag* mRNA. (**F**) Expression level of *Zbp1* mRNA. (**G**) Immunoblots of phospho-Mlkl, Mlkl, and Gapdh; protein amounts of phospho-Mlkl relative to Mlkl are shown (*n* = 5 in each group). (**H**) Immunoblot analysis of Mlkl in cytoplasmic and membrane extracts. Nonreducing and reducing SDS-PAGE were used to detect oligomerized and total Mlkl forms, respectively. (**I**) Representative images of intestinal organoids before and after treatment with ethanol (50 mM). (**J**) Lactate dehydrogenase (LDH) assay of supernatants was performed to measure cytotoxicity. Results were generated from 3 (**B**) or 2 (**C–J**) technical replicates. *P* values among groups were determined by Mann-Whitney U test (**B–D**) or 1-way ANOVA with Tukey’s post hoc test (**E–G** and **J**). Results are expressed as mean ± SEM. FMT, fecal microbiota transplantation. *P < 0.05, **P < 0.01, ***P < 0.001, ****P < 0.0001. AU, arbitrary units.

To determine whether mice, which also carry ERVs (22), can serve as a model to better understand the role of ERVs in the pathogenesis of alcohol-related liver disease, we employed 3 different ethanol-induced liver disease models: (a) chronic-binge ethanol feeding (23, 24) in microbiota-humanized mice (colonization of germ-free mice with feces from patients with alcohol-associated hepatitis is known to exacerbate ethanol-induced liver disease, compared with feces from conventional mice, (24); Figure 2A and Supplemental Figure 2A); (b) chronic-binge ethanol feeding in conventional mice (23) (Supplemental Figure 2D); and (c) intragastric hybrid feeding model of ethanol, which combines ethanol and Western diet feeding (25) (Supplemental Figure 2G). Expression of the murine betaretrovirus mouse mammary tumor virus (MMTV) transcripts *env* and *gag* was induced in all 3 murine models along the small and large intestine after ethanol feeding compared with mice on an isocaloric diet without ethanol (Figure 2B and Supplemental Figure 2, E and H). Similarly, transcription of ERVs derived from gammaretroviral murine leukemia virus (MLV), including ecotropic MLV (*eMLV*), polytropic MLV (*pMLV*), and modified polytropic MLV (*mpMLV*), was significantly induced in the ileum of ethanol-fed microbiota-humanized mice (Supplemental Figure 2B). Notably, ERVs were not induced by ethanol in the liver in any of the 3 models.

To determine whether increased ERV expression was detected by the host, we assayed the activation of Zbp1- and Mlkl-mediated necroptosis. Phosphorylated Mlkl assembles into oligomers, some of which translocate to the plasma membrane and induce necroptosis by causing rupture of the cell membrane (26). IECs isolated from ethanol-fed mice showed an increase in Zbp1 mRNA (Figure 2C) and protein expression and increased Mlkl phosphorylation (Figure 2D). Hepatic *Zbp1* expression was not changed in mice subjected to the chronic-binge ethanol feeding (Supplemental Figure 2, C and F), but it was induced in mice following intragastric hybrid feeding of ethanol (Supplemental Figure 2I) indicating that other factors besides ERVs can also stimulate Zbp1 expression. To extend these findings, we treated mouse intestinal organoids with different concentrations of ethanol (ranging from 10 to 50 mM) for 24 hours. Ethanol treatment led to a dose-dependent increase in *MMTV* *env* and *gag, eMLV*, *pMLV*, and *mpMLV* expression (Figure 2E and Supplemental Figure 3A); *Zbp1* expression (Figure 2F); increased phosphorylation of Mlkl (Figure 2G); increased translocation from cytosol to membrane and oligomerization of Mlkl (Figure 2H); and increased cell death (Figure 2, I and J). Ethanol did not increase apoptosis (cleaved caspase-3) or pyroptosis (cleaved gasdermin D) in intestinal organoids (Supplemental Figure 3B). These results might indicate that ethanol-induced ERVs signal through Zbp1 and Mlkl to induce necroptosis in IECs.

### Suppression of ERVs or inhibition of Mlkl reduces ethanol-induced necroptosis in intestinal organoids.

**Figure 3:**
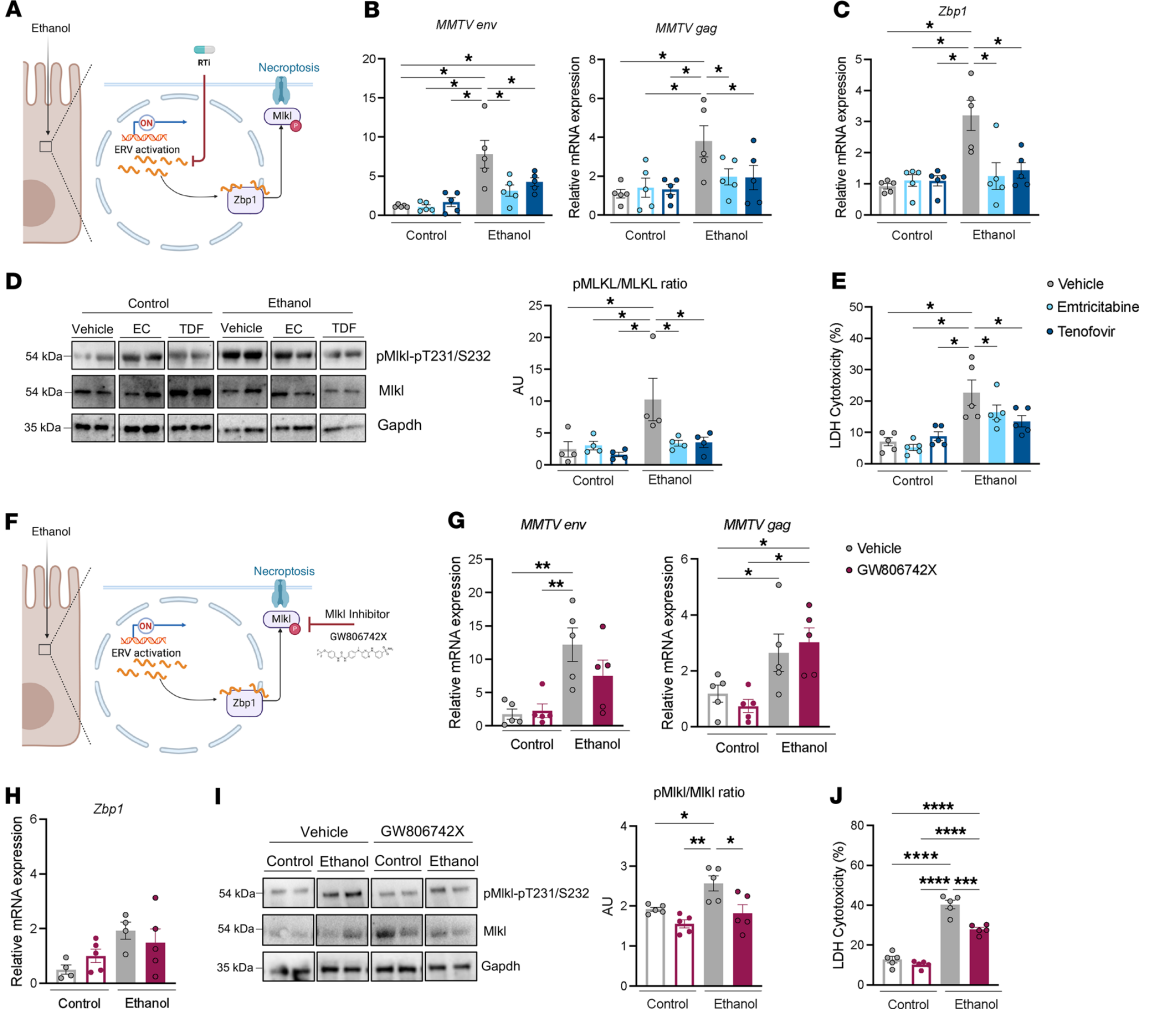
Ethanol-induced necroptosis is reduced by suppressing ERV expression or inhibiting Mlkl in intestinal organoids. (**A**) Mouse intestinal organoids were treated with the antiretroviral agents emtricitabine (EC; 100 μM) or tenofovir disoproxil fumarate (TDF; 100 μM) and incubated with ethanol (50 mM) for 24 hours. (**B**) Expression levels of mouse mammary tumor virus (*MMTV*) *env* and *gag* mRNA. (**C**) Expression level of *Zbp1* mRNA. (**D**) Immunoblots of phospho-Mlkl, Mlkl, and Gapdh; protein amounts of phospho-Mlkl relative to Mlkl are shown (*n* = 4 in each group). (**E**) Lactate dehydrogenase (LDH) assay of supernatants was performed to measure cytotoxicity. (**F**) Mouse intestinal organoids were treated with the Mlkl inhibitor GW806742X (2 μM) and incubated with ethanol (50 mM) for 24 hours. (**G**) Expression levels of *MMTV env* and *gag* mRNA. (**H**) Expression level of *Zbp1* mRNA. (**I**) Immunoblots of phospho-Mlkl, Mlkl, and Gapdh; protein amounts of phospho-Mlkl relative to Mlkl are shown (*n* = 5 in each group). (**J**) Lactate dehydrogenase assay of supernatant was performed to measure cytotoxicity. Results were generated from 3 (**B** and **C**) or 2 (**D**–**J**) technical replicates. *P* values among groups were determined by 1-way ANOVA with 2-stage step-up method of Benjamini, Krieger and Yekutieli test (**B–E**) or Tukey’s post hoc test (**G–J**). Results are expressed as mean ± SEM. RTi, reverse transcriptase inhibitor. *P < 0.05, **P < 0.01, ***P < 0.001, ****P < 0.0001. AU, arbitrary units.

We next treated intestinal organoids with the antiretrovirals emtricitabine or tenofovir for 24 hours and evaluated the effect on ethanol-induced necroptosis (Figure 3A). Treatment with emtricitabine or tenofovir led to a downregulation of ethanol-induced *MMTV env* and *gag* expression in intestinal organoids (Figure 3B), which was accompanied by reduced *Zbp1* expression (Figure 3C) and Mlkl phosphorylation (Figure 3D). In parallel, antiretroviral treatment resulted in a significant decrease in cytotoxicity following incubation with ethanol (Figure 3E). These results demonstrate that antiretroviral treatment reduces ethanol-induced ERV activation, activation of Zbp1/Mlkl signaling, and cell death. Since antiretroviral treatment did not reduce cell death back to the level of control treated cells, we cannot rule out that other stimuli, such as inflammatory cytokines, or ethanol metabolites, such as acetaldehyde, might contribute to a lesser extent to ethanol-induced cell death.

To further understand the role of ethanol in inducting necroptotic death in IECs, we investigated the effect of the Mlkl inhibitor GW806742X on ethanol-induced cell death (Figure 3F). Treatment of intestinal organoids with ethanol and GW806742X did not reduce ERV (*MMTV env* and *gag*) expression (Figure 3G) or *Zbp1* expression after 24 hours (Figure 3H). As expected, GW806742X blocked the ethanol-induced phosphorylation of Mlkl (Figure 3I). Importantly, treatment with GW806742X reduced ethanol-induced necroptosis in intestinal organoids (Figure 3J). We confirmed a decrease in ethanol-induced necroptosis following genetic knockdown of *Mlkl* in a mouse IEC line, MODE-K cells (Supplemental Figure 3, C and D). These results indicate that ethanol triggers necroptosis by induction of ERV expression and activation of the Mlkl signaling pathway in IECs.

### Overexpression of HERV-K induces necroptosis in ethanol-treated intestinal cells.

**Figure 4:**
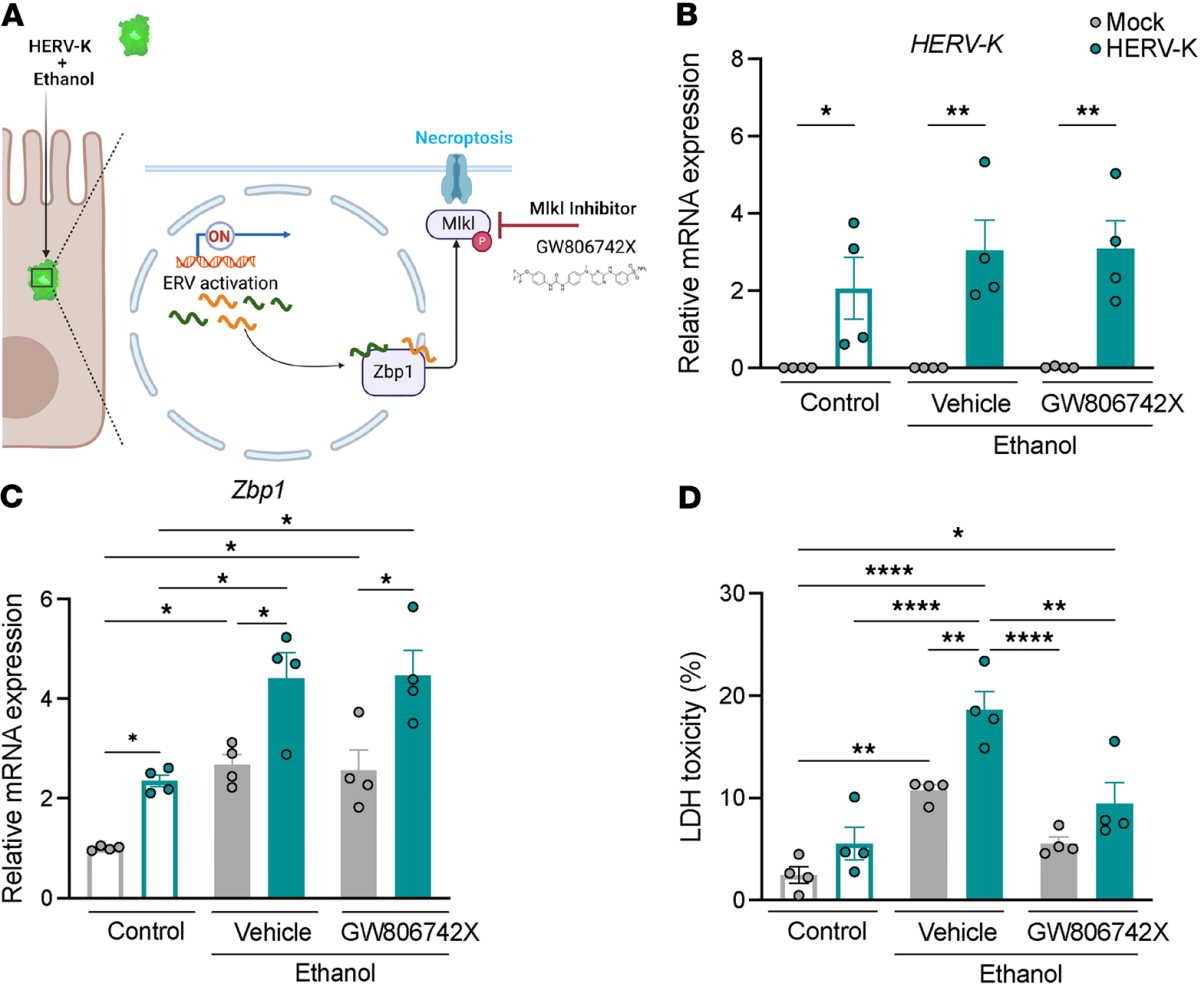
Overexpression of *HERV-K* induces necroptosis in ethanol-treated intestinal cells. (**A**) MODE-K cells were infected with a lentiviral control vector (Mock) or a lentivirus overexpressing human endogenous retroviruses K (*HERV-K*). Infected MODE-K cells were treated with 50 mM ethanol and the Mlkl inhibitor GW806742X (2 μM) for 24 hours. (**B**) Expression levels of *HERV-K* mRNA. (**C**) Expression level of *Zbp1* mRNA. (**D**) Lactate dehydrogenase (LDH) assay of supernatant was performed to measure cytotoxicity. (**B–D**) Results were generated from 4 technical replicates. *P* values among groups were determined by 1-way ANOVA with Tukey’s post hoc test. Results are expressed as mean ± SEM. *P < 0.05, **P < 0.01, ****P < 0.0001.

To assess whether HERVs are able to directly induce necroptosis, we overexpressed *HERV-K* in MODE-K cells in the presence or absence of ethanol (Figure 4, A and B). *HERV-K* overexpression significantly increased Zbp1 mRNA levels with and without ethanol (Figure 4C). *HERV-K* overexpression also increased cell death, although only in presence of ethanol, indicating that both *HERV-K* and ethanol are required for this effect (Figure 4D). Ethanol alone also triggered cell death, likely due to induction of mouse ERVs. Importantly, the effect of *HERV-K* and ethanol in cell death was almost completely attenuated by GW806742X, indicating that *HERV-K* and ethanol induce necroptotic form of cell death (Figure 4D).

### Antiretroviral treatment reduces ethanol-induced liver disease in microbiota-humanized mice.

**Figure 5:**
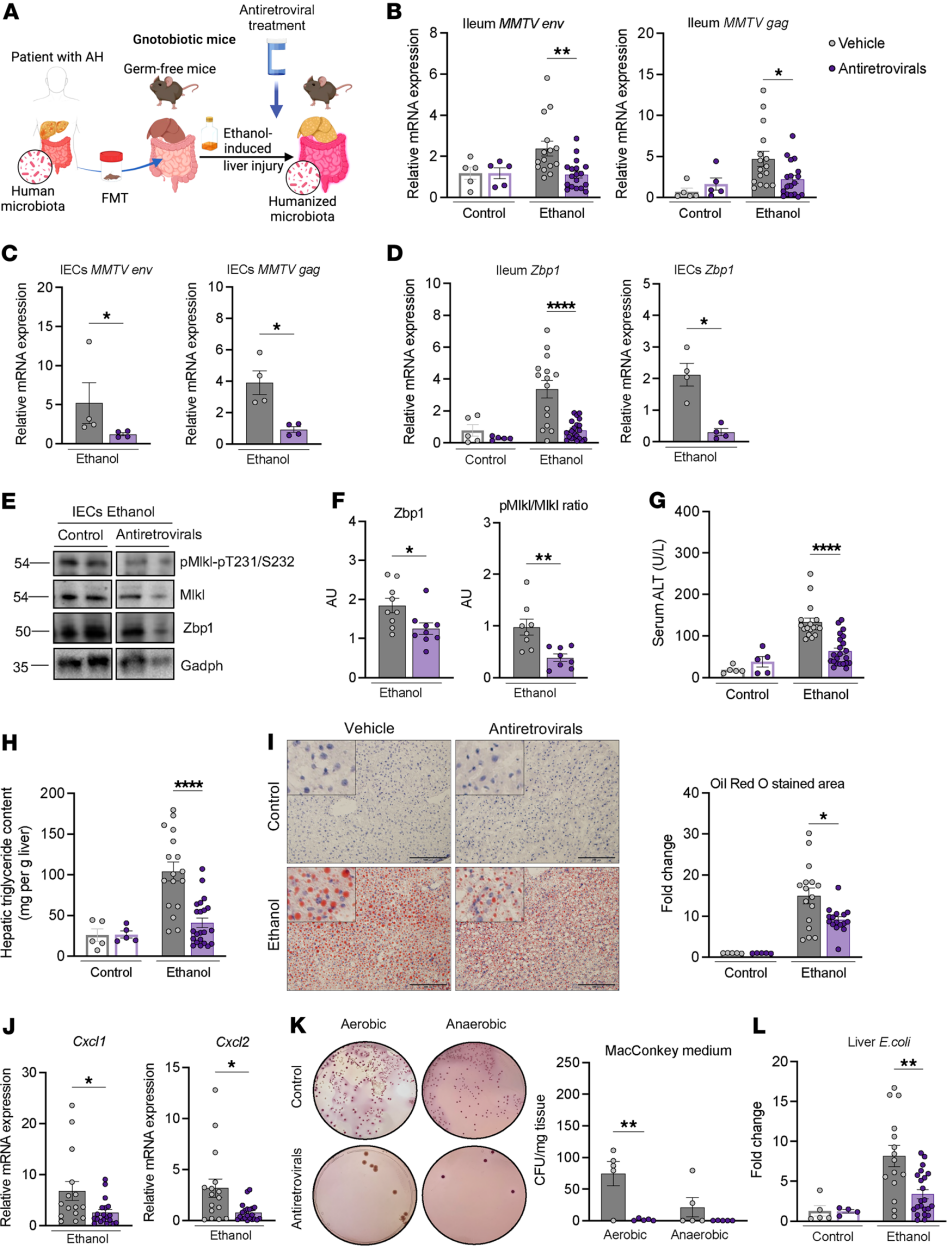
Antiretroviral treatment reduces ethanol-induced intestinal necroptosis and liver disease. (**A**) Germ-free C57BL/6 mice were colonized with fecal microbiota from patients with alcohol-associated hepatitis (AH) and treated with a combination of antiretrovirals (660 μM emtricitabine, 314 μM tenofovir, and 375 μM nevirapine) in the drinking water after second colonization and then in liquid diet. Gnotobiotic mice were fed oral isocaloric (control) or chronic-binge ethanol diets. (**B**) Levels of mouse mammary tumor virus (*MMTV*) *env* and *gag* mRNA in the ileum. (**C**) Levels of *MMTV env* and *gag* mRNA in intestinal epithelial cells (IECs) isolated from the small intestine. (**D**) Levels of *Zbp1* mRNA in the ileum and in intestinal epithelial cells isolated from the small intestine. (**E** and **F**) Intestinal epithelial cells were isolated from ethanol-fed mice, and immunoblots were performed for phospho-Mlkl, Mlkl, Zbp1, and Gapdh; protein amounts of Zbp1 relative to Gapdh and protein amounts of phospho-Mlkl relative to Mlkl are shown (*n* = 8 in each group). (**G**) Serum levels of ALT. (**H**) Hepatic triglyceride content. (**I**) Representative images of liver sections stained with Oil Red O (scale bars: 200 μm) and quantification of Oil Red O-stained area. (**J**) Hepatic levels of *Cxcl1* and *Cxcl2* mRNA. (**K**) Colony-forming units on MacConkey agar plates from the liver. (**L**) Hepatic *E*. *coli* abundance normalized to bacterial 16S, as measured by qPCR. (**B–L**) Results were generated from 2 technical replicates. *P* values among groups were determined by 1-way ANOVA with Tukey’s post hoc test (**B**; **D**, left; **G–I**; and **L**) or Mann-Whitney U test (**C**, **D**, right, **F**, **J**, and **K**). Results are expressed as mean ± SEM. *P < 0.05, **P < 0.01, ****P < 0.0001. AU, arbitrary units.

To further investigate the role of ERVs in ethanol-induced liver disease in vivo, we colonized germ-free mice with stool from patients with alcohol-associated hepatitis (24). Microbiota-humanized mice were administered a combination of antiretrovirals (emtricitabine, tenofovir, and nevirapine) (20) in a liquid diet and placed on a chronic-binge ethanol or isocaloric diet as a control, and ethanol-induced liver disease was studied (Figure 5A). No significant differences were observed in body weight or food intake (Supplemental Figure 4, A and B). Antiretroviral treatment reduced *MMTV env* and *gag* expression in the ileum and in IECs isolated from the small intestine of ethanol-fed mice (Figure 5, B and C). *MMTV* *env* and *gag* were not significantly induced in the liver following ethanol feeding (Supplemental Figure 4C). *Zbp1* gene expression was suppressed in the ileum and isolated IECs from the small intestine of mice subjected to the chronic-binge ethanol feeding model and given antiretroviral treatment (Figure 5D). Furthermore, Zbp1 protein levels and Mlkl phosphorylation were significantly reduced in isolated small IECs following ethanol feeding when treated with antiretrovirals (Figure 5, E and F), indicating less necroptotic cell death in IECs. Compared with vehicle-treated mice, mice fed ethanol and receiving antiretrovirals developed less liver injury, indicated by a lower level of serum alanine amino-transferase (Figure 5G), decreased hepatic steatosis (Figure 5, H and I), and reduced liver inflammation, with decreased expression of the inflammatory chemokines, chemokine (C-X-C motif) ligand-1 (*Cxcl1*) and *Cxcl2* (Figure 5J). Antiretrovirals also decreased the number of colony-forming units of Gram-negative bacteria that translocated from the gut to the liver after ethanol feeding (Figure 5K). Using qPCR on genomic DNA isolated from liver tissue, we show a significant reduction in hepatic *E*. *coli* in ethanol-fed mice after antiretroviral treatment (Figure 5L). No significant differences were observed in serum level of ethanol or the mRNAs encoding the two primary enzymes that metabolize ethanol in the liver, alcohol dehydrogenase 1 (*Adh1*) and cytochrome P450 family 2 subfamily E polypeptide 1 (*Cyp2e1*) (Supplemental Figure 4, D and E). Similarly, gene expression of *Cyp2e1* and *Adh1*, and of the acetaldehyde-metabolizing enzyme aldehyde dehydrogenase 2 (*Aldh2*) was not significantly different in the duodenum between the different groups of ethanol-fed mice (Supplemental Figure 4F). To compare the effect of antiretroviral treatment with a systemic Mlkl inhibitor, microbiota-humanized mice were placed on a chronic-binge ethanol diet and treated with the Mlkl inhibitor GW806742X by intraperitoneal injection. Mice receiving GW806742X had significantly less liver injury and steatosis as compared with ethanol-fed mice. Antiretroviral treated mice had slightly, but significantly lower serum alanine aminotransferase (ALT) levels as compared with GW806742X-administered mice. Steatosis was similar between ethanol-fed mice receiving antiretrovirals or GW806742X (Supplemental Figure 4, G and H).

These results indicate that antiretroviral therapy effectively attenuates ethanol-induced ERV activation, gut barrier dysfunction, and liver disease.

### Mice lacking Zbp1 in IECs are protected from ethanol-induced liver disease.

**Figure 6:**
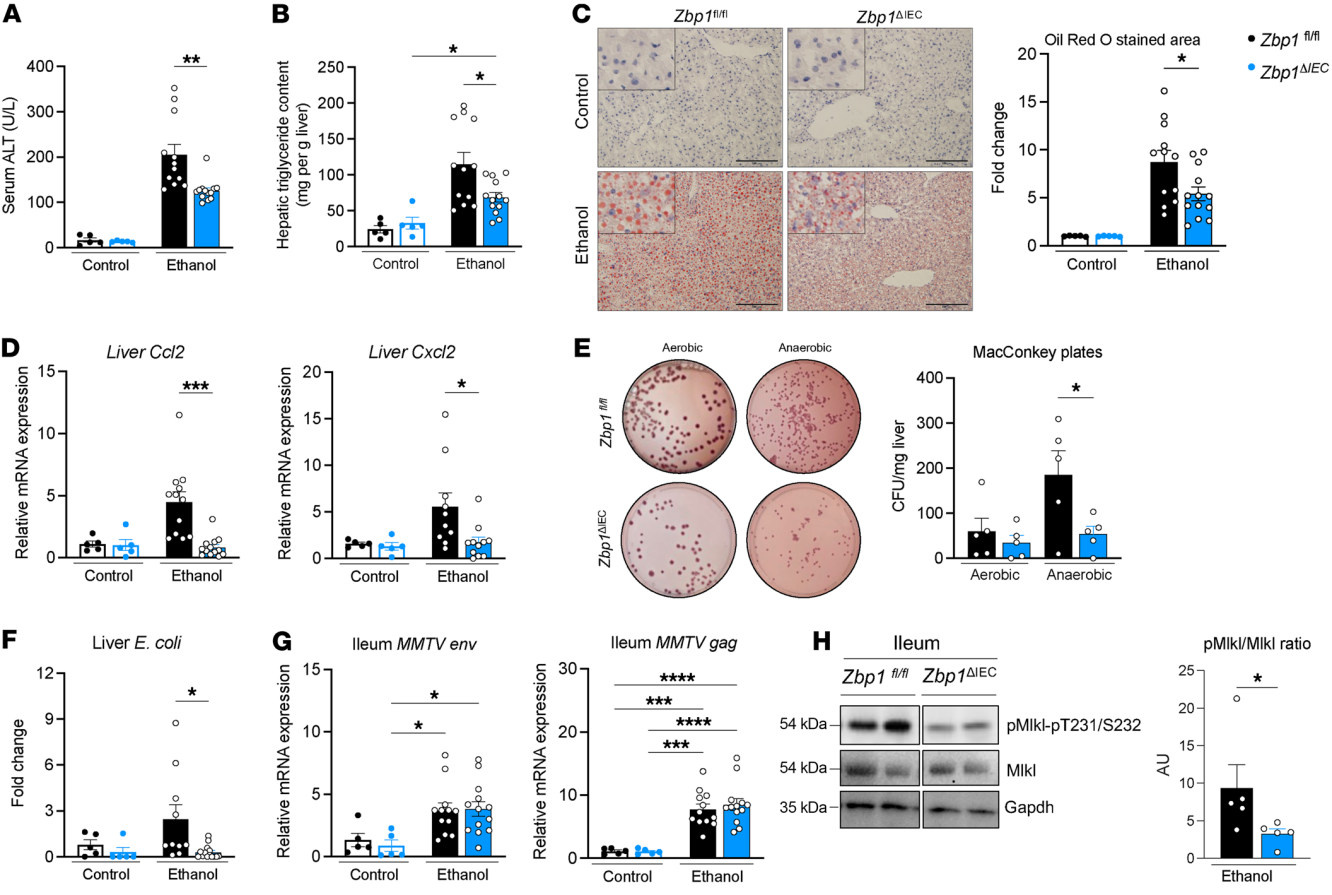
Deficiency of *Zbp1* in intestinal epithelial cells attenuates ethanol-induced intestinal necroptosis and liver disease. Mice lacking *Zbp1* in intestinal epithelial cells (*Zbp1*^ΔIEC^) and their *Zbp1^fl/fl^* littermate controls were fed oral isocaloric (control) or chronic-binge ethanol diets. (**A**) Serum levels of ALT. (**B**) Hepatic triglyceride content. (**C**) Representative images of liver sections stained with Oil Red O (scale bars: 200 μm) quantification of Oil Red O–stained area. (**D**) Hepatic levels of *Ccl2* and *Cxcl2* mRNA. (**E**) Colony-forming units on MacConkey agar plates from the liver. (**F**) Hepatic *E*. *coli* abundance normalized to bacterial 16S, as measured by qPCR. (**G**) Levels of *MMTV env* and *gag* mRNA in the ileum. (**H**) Immunoblots of phospho-Mlkl, Mlkl, and Gapdh; protein amounts of phospho-Mlkl relative to Mlkl are shown (*n* = 5 in each group). Results were generated from 2 technical replicates. *P* values among groups were determined by 1-way ANOVA with Tukey’s post hoc test (**A–D**, **F**, and **G**) or Mann-Whitney U test (**E** and **H**). Results are expressed as mean ± SEM. *P < 0.05, **P < 0.01, ****P < 0.0001.*P < 0.05, **P < 0.01, ***P < 0.001, ****P < 0.0001. AU, arbitrary units.

To further corroborate the role of *Zbp1* in mediating the effect of ERVs in the intestine, we used mice with an IEC-specific deletion of *Zbp1* (*Zbp1*^ΔIEC^). This was achieved by generating a *Zbp1* conditional knockout mouse by CRISPR/Cas-mediated genome engineering, which was crossed with a villin-Cre transgenic mouse (Supplemental Figure 5A). qPCR and immunoblot analysis confirmed loss of Zbp1 expression in the intestine and IECs but not in the liver of *Zbp1*^ΔIEC^ mice as compared with *Zbp1*^fl/fl^ littermates (Supplemental Figure 5, B–E). Compared with *Zbp1*^fl/fl^ mice, *Zbp1*^ΔIEC^ mice had less ethanol-induced liver injury (Figure 6A), steatosis (Figure 6, B and C), and inflammation (Figure 6D) following chronic-binge ethanol feeding without change in body weight or food intake (Supplemental Figure 5, F and G). Translocation of viable *E*. *coli* to the liver was suppressed in ethanol-fed *Zbp1*^ΔIEC^ mice (Figure 6, E and F). Absence of *Zbp1* in IECs did not affect ERV expression in the intestine (Figure 6G) or the liver (Supplemental Figure 5H) following ethanol feeding. *Zbp1*^ΔIEC^ mice fed an ethanol diet showed less activation and phosphorylation of Mlkl in the intestine (Figure 6H). Intestinal absorption of ethanol, expression of hepatic and duodenal genes involved in ethanol, and acetaldehyde metabolism were similar in all groups (Supplemental Figure 5, I–K).

To further support the importance of intestinal rather than hepatic Zbp1 in mediating the effect on ethanol-induced liver disease, we generated mice with specific deletions of *Zbp1* in Kupffer cells (*Zbp1*^ΔKC^) (Supplemental Figure 6, A and B) and hepatocytes (*Zbp1*^ΔHep^) (Supplemental Figure 6, H and I). *Zbp1*^ΔKC^ and *Zbp1*^ΔHep^ mice did not show significant differences in ethanol-induced liver disease compared with their respective *Zbp1*^fl/fl^ littermates (Supplemental Figure 6, C–G and J–N). Taken together, these findings demonstrate the critical role of intestinal epithelial Zbp1 in ethanol-induced liver disease.

## Discussion

Our results provide new insights into the pathogenesis of alcohol-associated liver disease. We show that alcohol triggers ERV expression in the intestine, which leads to activation of Zbp1 and induction of the necroptosis pathway in IECs. Consequently, the gut barrier becomes dysfunctional, allowing translocation of Gram-negative bacteria such as *E*. *coli* to the liver. Translocated bacteria in the liver are known to cause a progression of liver disease (27). And indeed, bacterial translocation and ethanol-induced liver disease are reduced by either suppression of ERVs using antiretrovirals or elimination of Zbp1 in IECs. Necroptosis is usually initiated in response to certain types of stress or infection, including viral infections (28). The necroptotic pathway controls infections by eliminating infected cells. In our study, ethanol exploits this pathway by inducing ERVs and stimulating innate immune responses, resulting in the elimination of IECs and disruption of the gut barrier.

Our data show that HERV expression is induced in the intestines and the livers of patients with alcohol use disorder. In line with our results, MMTV-like virus env sequences (an ortholog of the human betaretrovirus) were found in 50% of liver samples from patients with alcohol-associated cirrhosis but were not detected in normal liver (29). Consistent with the induction of hepatic HERVs in patients with alcohol-associated liver disease, ZBP1 protein expression is increased in the livers of patients with severe alcohol-associated hepatitis, with noncoding host-derived 5S rRNA pseudogene (RNA5SP) transcripts proposed as upstream activator (30). Ethanol causes epigenetic changes and remodels the chromatin by histone modification such as acetylation (31, 32). It is possible that ethanol influences ERV expression through chromatin remodeling, but this requires further research.

Interestingly, ERVs are not induced in the livers of mice fed ethanol, while HERVs are elevated in the livers of patients with alcohol use disorder. Several factors could contribute to these differences. Mouse ERVs and HERVs are specific to their host genomes (33). Mechanisms for controlling the expression of these genetic elements might be different between mice and humans. Alcohol use disorder often leads to progressive liver disease in patients, while mice are resistant to developing advanced liver disease following ethanol administration. Therefore, the differential ERV response to ethanol between mice and humans might affect susceptibility to liver disease. Further research is required to delineate the role of HERV expression in the liver.

The antiretroviral drugs tenofovir and emtricitabine are nucleotide/nucleoside reverse transcriptase inhibitors and inhibit transcription of viral RNA into DNA. Since *MMTV env* and *gag* mRNA are suppressed after antiretroviral treatment and since tenofovir and emtricitabine do not inhibit transcription of reactivated ERVs, it is possible that there are replicative intermediates of exogenous retroviruses or active retrotransposition resulting in the formation of viral RNA/cDNA hybrids. And indeed, resurrection of ERVs into infectious retroviruses has been described in immunodeficient mice (34). Nevirapine directly inhibits the reverse transcriptase enzyme and is specific for HIV, which is the reason it might have limited efficacy in our mouse model.

The precise mechanism linking necroptosis of IECs to bacterial translocation remains unknown. Reactivation of ERVs in the intestine caused by dysfunctional *Setdb1*, induces Zbp1-mediated necroptosis, which triggers intestinal inflammation (21). Necroptosis might create gaps in the epithelial barrier, allowing bacteria to translocate into the underlying tissue and subsequently to the liver.

Taken together, we uncovered a process by which alcohol leads to viral mimicry by inducing ERVs in IECs. This triggers Zbp1-mediated necroptosis in the intestinal epithelium and barrier disruption, causing bacterial translocation and progression of alcohol-associated liver disease.

## Methods

### Sex as a biological variable.

Male and female patients were used in this study. Male and female mice were used in this study.

### Patient cohorts.

Duodenal biopsies were obtained from 16 individuals without alcohol use disorder or alcohol-associated liver disease (individuals acting as controls) and 44 patients with alcohol use disorder and alcohol-associated liver disease at an alcohol withdrawal unit in Brussels, Belgium, where they underwent a detoxification and rehabilitation program. Demographic and laboratory characteristics of both individuals acting as controls and patients are detailed in Supplemental Table 1. Patients who were actively drinking until the day of admission, underwent transient elastography with controlled attenuation parameter (CAP) (Fibroscan, Echosens) at admission. Upper endoscopy with biopsies was performed on day 2 after admission. Exclusion criteria included the use of antibiotics or immunosuppressive medication in the 2 months before enrollment, diabetes, inflammatory bowel disease, known liver disease of any other etiology, and clinically significant cardiovascular, pulmonary, or renal comorbidities. Severity of liver disease was characterized using readily available clinical parameters. A median CAP value >250 dB/m was applied to diagnose steatosis (35), which was further confirmed by Doppler ultrasound. For distinguishing between nonprogressive (simple steatosis) and progressive disease (steatohepatitis or steatofibrosis), criteria included ALT and aspartate aminotransferase (AST) levels >40 U/L or a liver stiffness measurement (LSM) >7.8 kPa (36). Specific thresholds for liver stiffness were utilized to differentiate between stages of fibrosis: an LSM of 7.6 kPa for F1/F2 and 8.8 kPa for F2/F3 (37).

Patients with suspected significant fibrosis of ≥F2 on Fibroscan were routinely offered a transjugular liver biopsy. Initially, we used the cutoff of 7.8 kPa proposed by Nguyen-Khac et al. in 2008 (38). This cutoff was adapted to 9.0 kPa after publication of a meta-analysis by the same authors (39). Biopsy samples of ≥15 mm in length, including a minimum of 6 portal tracts, were considered suitable or when an experienced pathologist could establish the degree of fibrosis with certainty on a smaller sample. Twenty-seven patients consented to undergo a clinically indicated liver biopsy, which was performed on the third day of admission. Demographic and biochemical data are summarized in Supplemental Table 2. Significant fibrosis (≥F2 according to the Metavir system) was confirmed on histology in 19 patients whereas 8 patients were downgraded to F1. Liver samples from size-reduced liver grafts were used as controls. Histology revealed a normal appearance of the tissue. For ethical reasons and for protection of the donors’ confidentiality, no additional data are available for those samples.

### Mice.

To assess ERV expression in mice, we employed 3 mouse models. (a) Germ-free female and male C57BL/6 mice (age 8 weeks) were bred in the gnotobiotic facility at the University of California, San Diego, as described previously (40). Germ-free status of the animals was routinely confirmed by plating fecal homogenates on blood agars and incubation for up to 2 days at 37°C under both aerobic (room air) and anaerobic (AnaeroPack System, Mitsubishi) conditions. Stool from 5 different, HIV-negative patients with alcohol-associated hepatitis was used for fecal microbiota transplantation into germ-free mice. Mice were gavaged with 100 μL of stool samples (1 g stool dissolved in 30 mL Luria-Bertani medium containing 15% glycerol under anaerobic conditions), starting at an age of 4–5 weeks and repeated 2 weeks later. Two weeks after the second gavage, mice were placed on a chronic-binge ethanol diet (NIAAA model) or a control (isocaloric) diet as described previously (23). Mice were fed with a Lieber DeCarli diet, and the caloric intake from ethanol was 0% on days 1–5 and 36% from day 6 until the end of the study period. At day 16, mice were gavaged with a single dose of ethanol (5 g/kg body weight) in the early morning and euthanized 9 hours later. Pair-fed control mice received a diet with an isocaloric substitution of ethanol with dextrose. (b) Conventional male and female wild-type C57BL/6 mice were bred in our animal facility at the University of California, San Diego, and placed on a chronic-binge ethanol diet (NIAAA model) or control (isocaloric) diet as described previously (23). (c) The hybrid model of intragastric chronic ethanol combines exposure of ethanol and Western-type high-fat diet (25). This model was prepared by the Animal Core of the Southern California Research Center for ALPD and Cirrhosis. Briefly, C57BL/6 mice (age 8 weeks) were fed a solid Western diet high in cholesterol and saturated fat (1% w/w cholesterol, 21% calories lard, 17% calories corn oil) for 2 weeks before surgery to implant an intragastric feeding catheter. After recovery, mice were fed a liquid high-fat diet (36% calories corn oil) plus alcohol or isocaloric dextrose, plus weekly alcohol binge (3.5–5 g/kg) through the intragastric feeding tube. This intragastric feeding provided 60% of the required total daily caloric intake (614 calories/kg/d), and the remaining 40% of calories are consumed by ad libitum consumption of solid Western diet. The ethanol dose was increased to 27 g/kg/d over 51 days. Pair-fed control mice were given an isocaloric high-fat liquid diet.

To assess the therapeutic efficacy of antiretroviral treatment, oral treatment with a combination of emtricitabine (Cipla; 660 μM), tenofovir (Camber Pharmaceutical; 314 μM) and nevirapine (Aurobindo; 375 μM) was given (20). Antiretrovirals were added to the drinking water following the second fecal matter transplantation into germ-free mice from 4 different, HIV-negative patients with alcohol-associated hepatitis. Antiretrovirals were also included in the liquid diet at the start of the isocaloric feeding (day 0). Diets were replaced every third day. Male and female mice were placed on a chronic-binge ethanol diet (NIAAA model) or control (isocaloric) diet 2 weeks after the second gavage as described above (23). A subset of mice was treated with the Mlkl inhibitor GW806742X at a dose of 2 mg/kg by intraperitoneal injections 3 times a week while being subjected to the NIAAA model, using 10% dimethyl sulfoxide in saline as vehicle.

*Zbp1*-floxed mice were generated using CRISPR/Cas9 technology by Cyagen Biosciences. Briefly, guide RNAs targeting the *Zbp1* gene were designed, and a donor vector with loxP sites was coinjected with Cas9 mRNA into fertilized mouse eggs. Exons 2–3 were selected as conditional knockout regions. F0 founders were identified through PCR and sequence analysis, and successful founders were bred with wild-type mice for germline transmission. F1 animals were generated and confirmed by genotyping. Molecular characterization verified the presence of the loxP-flanked *Zbp1* allele. Mice lacking *Zbp1* in IECs (*Zbp1*^ΔIEC^) were generated by crossing *Zbp1*^fl/fl^ mice with IEC-specific *villin*-Cre mice (41). Mice lacking *Zbp1* in Kupffer cells (*Zbp1*^ΔKC^) were generated by crossing *Zbp1*^fl/fl^ mice with Kupffer cell–specific *Clec4f-Cre* mice (42). Mice lacking *Zbp1* in hepatocytes (*Zbp1*^ΔHep^) were generated by crossing *Zbp1*^fl/fl^ mice with *albumin-Cre* mice (43). Female and male *Zbp1*^ΔIEC^, *Zbp1*^ΔKC^, and *Zbp1*^ΔHep^ mice and their respective control *Zbp1*^fl/fl^ littermate mice (age, 8 weeks) were placed on a chronic-binge ethanol diet (NIAAA model) or control (isocaloric) diet as described above (23).

### Epithelial cell isolation.

The intestine was removed from mice, rinsed with cold PBS, opened longitudinally, and cut into pieces of 1 cm length. Pieces were placed into ice-cold RPMI1640 medium (Invitrogen) with 5% FCS containing DTT (1 mM). After vigorous shaking, the supernatant was discarded, and the tissue was incubated for 20 minutes in RPMI1640 with 5% FCS containing EDTA (1 mM) at 37°C with shaking (250 rpm). IECs were collected from supernatant.

### Overexpression of HERV-K.

The plasmids used for HERV-K overexpression have been described previously (44). In brief, a 785 bp portion of *HERV-K* (corresponding to positions 7,291–8,076 bp in GenBank accession number AF074086.2) was subcloned into a lentiviral shuttle vector (FUGW), downstream of a nucleus-targeting GFP (NLS-GFP) reporter gene under the control of a strong synthetic CAG promoter. Lentiviral particles were produced and packaged by Gene Transfer, Targeting, and Therapeutics Core at the Salk Institute for Biological Studies, La Jolla, California, USA. The following lentivirus constructs were used: BL-1161 (CAG-NLS-GFP-P2A, empty control vector) and BL-1177 (CAG-NLS-GFP-HML2(TLR), expressing the 785 nt HERV-K sequence). For HERV-K overexpression, lentiviral constructs were transduced into MODE-K cells using polybrene (Invitrogen). Infected MODE-K cells were selected with Zeocin (Invitrogen) for 2 weeks and subsequently treated with 50 mM ethanol in the presence or absence of the Mlkl inhibitor GW806742X (2 μM; MedChemExpress) for 24 hours.

### siRNA-mediated knockdown of Mlkl.

MODE-K cells were transfected with specific small-interference RNA (siRNA) to silence the expression of Mlkl (siGENOME SMARTPool, M-005326-00-0005, Dharmacon) or nontargeting siRNA (siGENOME Non-Targeting siRNA Pool, D-001206-13-05, Dharmacon) for the control group. The transfection was performed using Lipofectamine 3000 Transfection Reagent (L3000008, Invitrogen) as follows: 0.1 nmol of each siRNA diluted in 300 μL optiMEM (31985070, Life Technologies) was mixed with 15 μL Lipofectamine 3000 diluted in 300 μL optiMEM; the mixture was added into each well of 24-well plates. After 8 hours, the medium was replaced with fresh growth medium. 24 hours after transfection, cells were exposed to 100 mM ethanol. 48 hours after transfection, supernatant was collected for LDH measurement, and cells were collected to check the efficiency of silencing by quantitative PCR.

### Human and mouse enteroids isolation and culture.

Human colon biopsies were preserved in 500 μL medium (DMEM F-12 with 10% FBS, 200 mM glutamax, and penicillin/streptomycin) containing ROCK inhibitors (Y-27632, 10 μM and A-83-01, 10 μM) on ice and then immediately processed to generate intestinal organoids as described previously (45). Briefly, biopsies were washed with ice-cold DPBS (Gibco) and cut using 2 scalpels in a sterile petri dish. Tissues were incubated at 37°C with collagenase type I solution (2 mg/mL) for 30 minutes with intermittent shaking and then filtered through a 70 μm strainer into a 10 mL conical tube. Isolated crypts were embedded in 40 μL Matrigel (CLS356231-1EA, Corning/Sigma) in a 24-well plate and cultured in a modified form of medium.

Small intestinal organoids (enteroids) were generated from wild-type C57BL/6 mice as described previously (46). Briefly, crypts were collected from the mouse small intestine after 30-minute incubation in PBS (pH 7.4) containing 2 mM EDTA at 4°C. Enteroids were plated in Matrigel (BD Bioscience) and maintained in DMEM/F12 (Life Technologies) containing ROCK inhibitors (Y-27632, 10 μM and A-83-01, 10 μM) and Fc-conditioned medium (the Rspo1-Fc–expressing cell line was a gift from Calvin Kuo, Stanford University, Stanford, California, USA). Cultures were passaged every 7–12 days and were typically split in a 1:6–1:8 ratio. To passage the 3-dimensional human organoid cultures, organoids were treated with trypsin. Following centrifugation, the pellet was resuspended in Matrigel, plated at 40 μL per well in a 24-well plate, incubated at 37°C, 5% CO_2_ for 10–20 minutes, and then 500 μL conditioned medium was added to each well.

Intestinal organoids from mice or humans were treated with freshly prepared solutions of ethanol (Sigma-Aldrich, 54965) with concentrations ranging from 10 mM to 50 mM for 24 hours. GW806742X (2 μM; MedChemExpress) was added to organoid cultures for 24 hours to inhibit Mlkl. To assess the therapeutic efficacy of antiretroviral treatment, intestinal organoids were treated with emtricitabine or tenofovir disoproxil fumarate at a final concentration of 100 μM for 24 hours.

### Primary mouse hepatocytes and Kupffer cells.

Mouse hepatocytes were isolated from C57BL/6 mice as described previously (47). Hepatocyte viability was consistently >85% as determined by trypan blue staining (Thermo Fisher Scientific). Hepatocytes were collected directly after harvesting for further experiments. Kupffer cells were isolated as described previously (27). Briefly, Kupffer cells were isolated from the 3-layer discontinuous density centrifugation gradient with 8.2% (wt/vol) and 14.5% (wt/vol) Nycodenz (Axis Shield). Kupffer cells were collected from the second layer. Cells were seeded into 6-well plates (1.5 × 10^6^ cells per well) and incubated for 2 days. RPMI medium containing 10% FBS and antibiotics was used for Kupffer cells.

### DNA extraction from mouse liver and feces.

DNA was isolated from mouse livers as described previously (48). Briefly, samples were resuspended in PBS and digested with 20 μL RNAse A (100 mg/mL) and 10 μL proteinase K (20 mg/mL) at 55°C for 1 hour. Each suspension was then transferred to individual Qbiogene lysing matrix B tubes and vortexed using a FastPrep FP120 instrument (Savant). The lysate was extracted twice using phenol/chloroform/isoamyl alcohol, and DNA was precipitated, washed with ethanol, and resuspended in TE buffer (Fisher Scientific, 12-090-015).

### Bacterial cultures of mouse liver.

Translocation of viable bacteria was assessed by culturing liver homogenates (49). Liver tissues (~100 mg) were aseptically dissected, homogenized in 1 mL of 0.05% NP-40 solution, and incubated at 37°C for 3 hours. After incubation, samples were washed with PBS, centrifuged at 3,500*g* for 10 minutes, and resuspended in 2 mL PBS. For culture, 100 μL of the liver homogenate was plated on MacConkey agar plates and incubated overnight at 37°C under aerobic and anaerobic conditions. Colony counts were performed.

### Biochemical analysis.

Serum levels of ALT were measured at 340 nm using ALT (SGPT) Kinetic (TECO Diagnostics). Hepatic triglyceride levels were measured using the Triglyceride Liquid Reagents Kit (Pointe Scientific). Levels of ethanol were measured using the Ethanol Assay Kit (BioVision). Cell cytotoxicity was assessed using Pierce LDH Cytotoxicity Detection Kit (Thermo Fisher Scientific).

### Real-time quantitative PCR.

RNA was extracted from liver and intestinal tissues using Trizol (Invitrogen). RNA was digested with DNase using the DNA-free DNA removal kit (Ambion), and cDNAs were generated using a high-capacity cDNA reverse transcription kit (Applied Biosystems). Primer sequences for mouse genes were originally obtained from the NIH qPrimerDepot. All primers used in this study are listed in Supplemental Table 3. qPCRs for human and mouse gene expression, amplification of genomic bacterial DNA and single colonies were run with Sybr Green (Bio-Rad Laboratories) using an ABI StepOnePlus real-time PCR system. The expression levels of the gene of interest in mouse and human samples were normalized to the total amount of the housekeeping gene 18S. Additionally, bacterial PCR from liver samples was normalized using the 16S gene.

### Oil Red O staining.

To determine lipid accumulation, liver sections were embedded in OCT (Tissue-TekR) compound. 5 μm frozen sections were then cut and stained with Oil Red O (Sigma-Aldrich). Four representative microphotographs of each animal at ×20 were taken with a BX51 Olympus microscope equipped with a DP72 Olympus digital camera, using Image J 2.9.0/1.53t software to quantify lipids (red area) from all Oil Red O–stained sections. Data are expressed as percentage of the control group.

### Immunoblot analyses.

Proteins were extracted and homogenized in 200 μL RIPA buffer (Thermo Scientific) with phosphatase inhibitors (Sigma-Aldrich) and protease inhibitors (Sigma-Aldrich) as described previously (50). Total protein from intestinal organoids was extracted as described previously (51). For analysis of Mlkl in the subcellular compartments, the Subcellular Protein Fractionation Kit for Cultured Cells (Thermo Scientific, 78840) was used, subjecting whole organoid lysate to Cytoplasmic Extraction Buffer (Thermo Scientific, 78840) and Membrane Extraction Buffer (Thermo Scientific, 78840) to obtain the cytoplasmic and membrane extracts, respectively. The proteins were resolved on polyacrylamide gels by SDS-PAGE (Bio-Rad) under reducing or nonreducing conditions and then transferred to polyvinylidene difluoride membranes (Bio-Rad). Immunoblot analysis was performed using anti-pMlkl antibody (1:1,000) (Cell Signaling, 37333), anti-Mlkl (1:1,000) (Millipore, MABC604), anti-Zbp1 (1:1,000) (Adipogen Life Science, AG-20B-0010-C100), anti-caspase-3 (1:1,000) (Cell Signaling, 9662), anti-cleaved caspase-3 (1:1,000) (Cell Signaling, 9664), anti-gasdermin D (cleaved and uncleaved) (1:1,000) (Thermo Fisher, MA5-44666), anti-Vdac (1:1,000) (Cell Signaling, 4866T), and anti-Gapdh (1:5,000) (GeneTex, GT239/ GTX627408). Protein levels were normalized to Gapdh for each sample and expressed as AU in relation to the control group. Efficiency of fractionation was evaluated via Gapdh for cytoplasmic extracts and Vdac for membrane extracts. Densitometry was done using Image Lab 2.0 software (Bio-Rad). All uncropped immunoblots are shown in Supplemental Figure 7.

### Statistics.

Human data were expressed as median and interquartile range for each continuous outcome, if not stated otherwise. Continuous variables were compared using Mann-Whitney test. Categorical variables were compared using the Pearson’s χ^2^ test. Spearman’s correlation was employed for correlation analysis. Results of the mouse studies are expressed as mean ± SEM. For mouse studies, significance between 2 groups was assessed by Mann-Whitney test, and significance between multiple groups was assessed by 1-way ANOVA with Tukey’s post hoc test or 2-stage step-up method of Benjamini, Krieger and Yekutieli. A *P* value of less than 0.05 was considered to be statistically significant. Statistical analyses were performed using R statistical software (R version 1.3.1093, 2020 the R Foundation for Statistical Computing) and GraphPad Prism v8.4.3.

### Study approval.

Written informed consent was obtained from all patients and individuals acting as controls, and the study protocol received approval from the Ethics Committee of the Université Catholique de Louvain in Brussels. Colon biopsies were obtained from patients during a clinically indicated colonoscopy at the VA San Diego Healthcare System. Written informed consent was obtained from all individuals prior to the procedure. The study was approved by an institutional review board at the VA San Diego Healthcare System and at the University of California, San Diego.

All animal studies were reviewed and approved by the Institutional Animal Care and Use Committees of the University of California, San Diego, and the University of Southern California. Animal use adhered to the guidelines in the most recent edition of the *Guide for the Care and Use of Laboratory Animals* (National Academies Press, 2011) and the *Guidelines for the euthanasia of animals* by the American Veterinary Medical Association. Sample size estimation was based on previous studies, and animals were randomly allocated to experimental groups. No animals were excluded from the analysis.

### Data availability.

Raw data are listed in the Supporting Data Values file.

## Author contributions

NC was responsible for the acquisition, analysis, and interpretation of data and writing the manuscript. MFF was responsible for the acquisition, analysis, and interpretation of data and writing the manuscript. WW, TY, FRT, AE, ASM, SM, and YW provided assistance with data acquisition. RGC provided assistance with human and mouse intestinal organoids. YM, LE, and HT provided assistance with conventional and gnotobiotic mouse studies. XZ provided assistance with statistical analysis. DEF and CL provided scientific advice and technical support. TT and SL provided the lentiviral vector and technical support. PS was responsible for collection of human samples. BS was responsible for the study concept and design, study supervision, and editing the manuscript. All authors reviewed and edited the manuscript.

## Supplementary Material

Supplemental data

Unedited blot and gel images

Supporting data values

## Version 1 05/13/2025

In-Press Preview

## Version 2 07/01/2025

Electronic publication
