# Supplementary material for: Activation of intestinal endogenous retroviruses by alcohol exacerbates liver disease
Source: J Clin Invest. 2025 May 13;135(13):e188541. doi: 10.1172/JCI188541 (PMC12208555; doi:10.1172/JCI188541)
Supplement: Supplemental data [file jci-135-188541-s258.pdf]

Supplementary materials to the manuscript:

Activation of intestinal endogenous retroviruses by alcohol exacerbates liver disease

Noemí Cabré[1#], Marcos F. Fondevila[1#], Wenchao Wei[1], Tomoo Yamazaki[1,2], Fernanda Raya Tonetti[1], Alvaro Eguileor[1], Ricard Garcia-Carbonell[3], Abraham S. Meijnikman[1], Yukiko Miyamoto[1], Susan Mayo[1], Yanhan Wang[1], Xinlian Zhang[4], Thorsten Trimbuch[5], Seija Lehnardt[6], Lars Eckmann[1], Derrick E. Fouts[7], Cristina Llorente[1], Hidekazu Tsukamoto[8,9], Peter Stärkel[10] and Bernd Schnabl[1,11]

[1]Department of Medicine, University of California San Diego, La Jolla, CA, USA
[2]Department of Medicine, Division of Gastroenterology and Hepatology, Shinshu University School of Medicine, Matsumoto, Japan
[3]Department of Molecular Medicine, The Scripps Research Institute, La Jolla, CA, USA
[4]Division of Biostatistics and Bioinformatics, Herbert Wertheim School of Public Health and Human Longevity Science, University of California San Diego, La Jolla, CA, USA
[5]Cluster of Excellence, NeuroCure, Charité Viral Core Facility, Charité – Universitätsmedizin Berlin, corporate member of Freie Universität Berlin, Humboldt-Universität zu Berlin, and Berlin Institute of Health, Berlin, Germany
[6]Institute of Cell Biology and Neurobiology, Charité–Universitätsmedizin Berlin, corporate member of Freie Universität Berlin, Humboldt-Universität zu Berlin, and Berlin Institute of Health (BIH), Berlin, Germany

[7]J. Craig Venter Institute, Rockville, MD, USA

[8]Department of Pathology, Keck School of Medicine of the University of Southern California, Los Angeles, CA, USA

[9]Department of Veterans Affairs Greater Los Angeles Healthcare System, Los Angeles, CA, USA

[10]Department of Hepatology and Gastroenterology, St. Luc University Hospital, Catholic University of Louvain, Brussels, Belgium

[11]Department of Medicine, VA San Diego Healthcare System, San Diego, CA, USA

[#]equal contribution

Correspondence: Bernd Schnabl, M.D., Department of Medicine, University of California San Diego, MC0063, 9500 Gilman Drive, La Jolla, CA 92093, Phone 858-822-5311, Fax 858-246-1788, Email beschnabl@ucsd.edu

Table of contents

Supplemental Figure 1……………………………………………………………………..4
Supplemental Figure 2……………………………………………………………………..6
Supplemental Figure 3……………………………………………………………………..8
Supplemental Figure 4……………………………………………………………………10
Supplemental Figure 5……………………………………………………………………12
Supplemental Figure 6……………………………………………………………………14
Supplemental Figure 7……………………………………………………………………16
Supplemental Table 1……………………………………………………………………18
Supplemental Table 2…………………………………………………………………….20
Supplemental Table 3…………………………………………………………………….22
References…………………………………………………………………………………24

Supplemental Figure 1

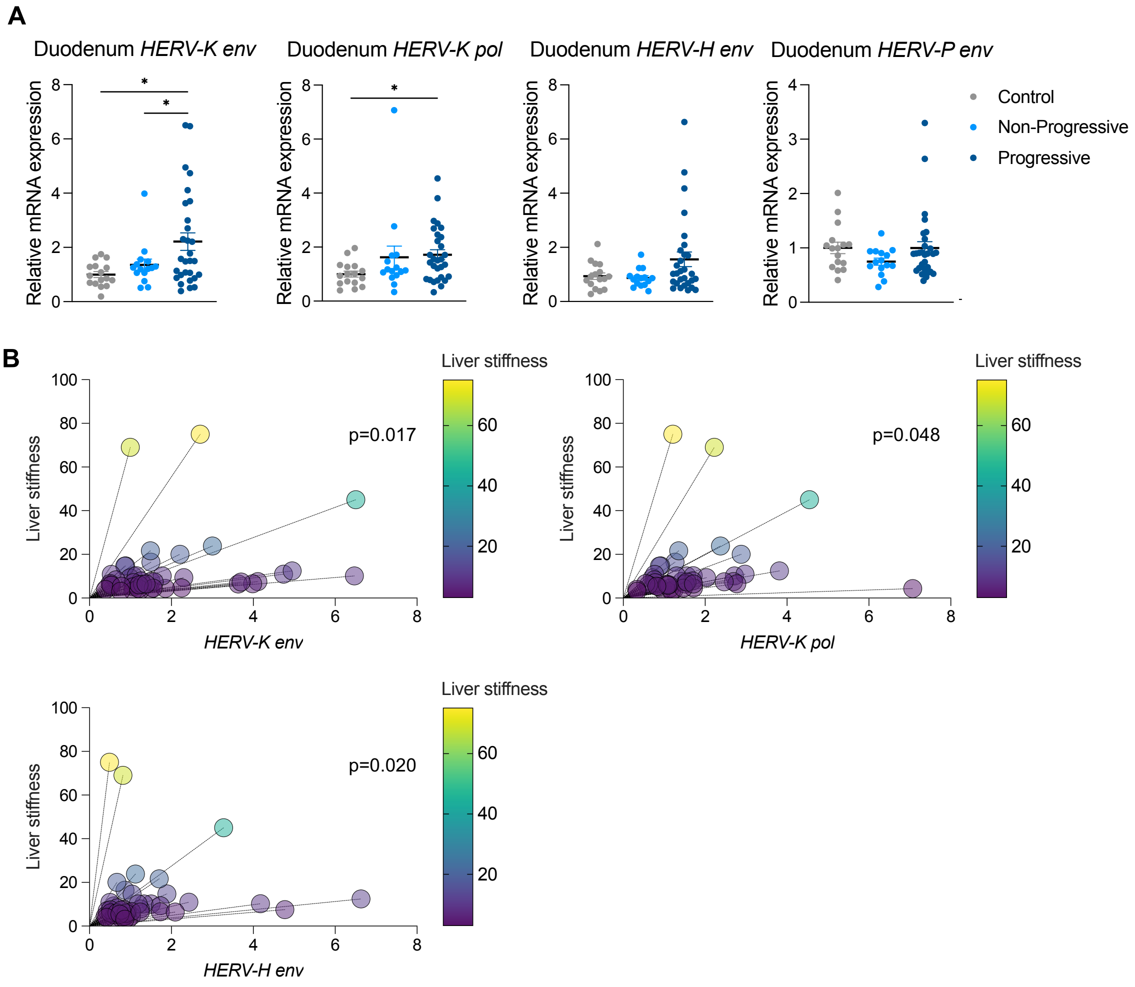

Supplemental Figure 1. Expression of Human Endogenous Retroviruses (HERVs) in duodenal biopsies of patients with alcohol-associated liver disease correlates with disease severity

(a) Duodenal biopsies were obtained from subjects without alcohol use disorder or alcohol-associated liver disease (controls; n=16) and patients with alcohol use disorder (n=44), and qPCR was performed to measure mRNA expression of Human Endogenous Retroviruses (HERVs). Expression levels of HERVs in patients with progressive liver disease compared to those with non-progressive alcohol-associated liver disease. **(b)** Correlation analysis between HERV expression and liver stiffness in patients with alcohol use disorder. P values among groups were determined by one-way analysis of variance (ANOVA) with Tukey's post hoc test **(a)** or Spearman correlation **(b)**. Results are expressed as mean ± SEM. $^{*}P < 0.05$

Supplemental Figure 2

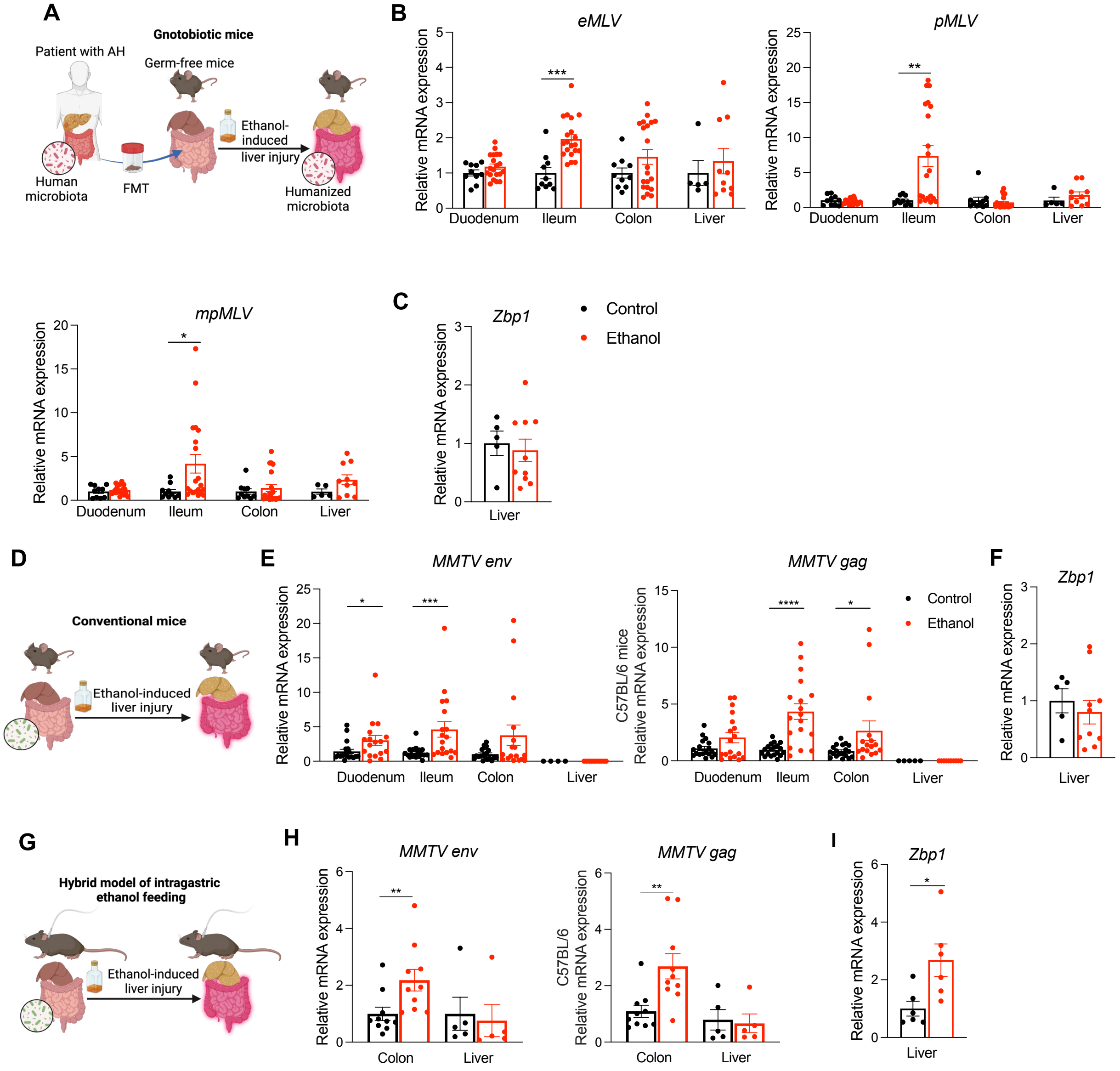

Supplemental Figure 2. Ethanol feeding induces ERV expression in the intestine of mice

(a–c) Germ-free C57BL/6 mice were colonized with stool from patients with alcohol-associated hepatitis (AH) and fed oral isocaloric (control) or chronic–binge ethanol diets. **(b)** Intestinal levels of ecotropic murine leukemia virus (*eMLV*)*,* polytropic MLV (*pMLV*) and modified polytropic MLV (*mpMLV*) mRNA. **(c)** Hepatic levels of *Zbp1* mRNA. **(d–f)** Conventional C57BL/6 mice were fed oral isocaloric (control) or chronic–binge ethanol diets. **(e)** Intestinal and hepatic levels of mouse mammary tumor virus (MMTV) *env* and *gag* mRNA. **(f)** Hepatic levels of *Zbp1* mRNA. **(g–i)** Conventional C57BL/6 mice were subjected to the hybrid model of intragastric chronic ethanol feeding. **(h)** Intestinal and hepatic levels of *MMTV env* and *gag* mRNA. **(i)** Hepatic levels of *Zbp1* mRNA. Results were generated from three **(b–c, e–f)** or two **(h–i)** technical replicates. P values among groups were determined by Mann-Whitney U test. Results are expressed as mean ± SEM. $^*P < 0.05$, $^{**}P < 0.01$, $^{***}P < 0.001$, $^{****}P < 0.0001$.

Supplemental Figure 3

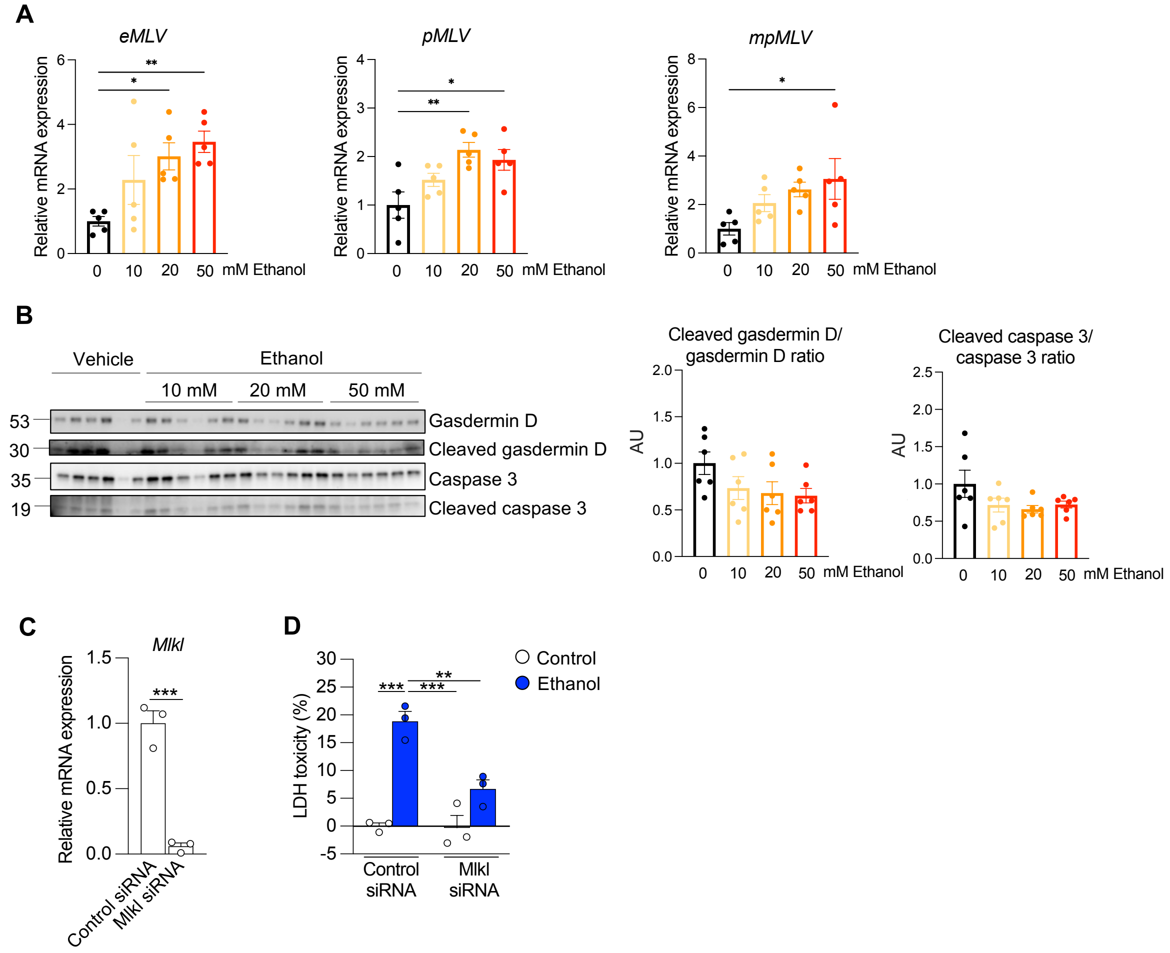

Supplemental Figure 3. Ethanol-induced necroptosis is reduced by silencing Mlkl in intestinal organoids

(a) Mouse intestinal organoids were incubated with ethanol (0, 10, 20, and 50 mM) for 24 hrs. Expression levels of ecotropic murine leukemia virus (*eMLV*), polytropic MLV (*pMLV*) and modified polytropic MLV (*mpMLV*) mRNA. **(b)** Mouse intestinal organoids were incubated with ethanol (0, 10, 20 and 50 mM) for 24 hrs. Immunoblots of gasdermin D, cleaved gasdermin D, caspase 3 and cleaved caspase 3; protein amounts of cleaved gasdermin D to gasdermin D, and cleaved caspase 3 to caspase 3. **(c–d)** MODE-K cells transfected with control siRNA or Mlkl siRNA. **(c)** *Mlkl* mRNA expression 48hrs after transfection. **(d)** Lactate dehydrogenase (LDH) toxicity following incubation with ethanol for 24h. Results were generated from two **(a–b)** or three **(c–d)** technical replicates. P values among groups were determined by one-way analysis of variance (ANOVA) with Tukey's post hoc test **(a–b, d)** or Mann-Whitney U test **(c)**. Results are expressed as mean ± SEM. AU, arbitrary units. *$P < 0.05$, **$P < 0.01$, ***$P < 0.001$.

Supplemental Figure 4

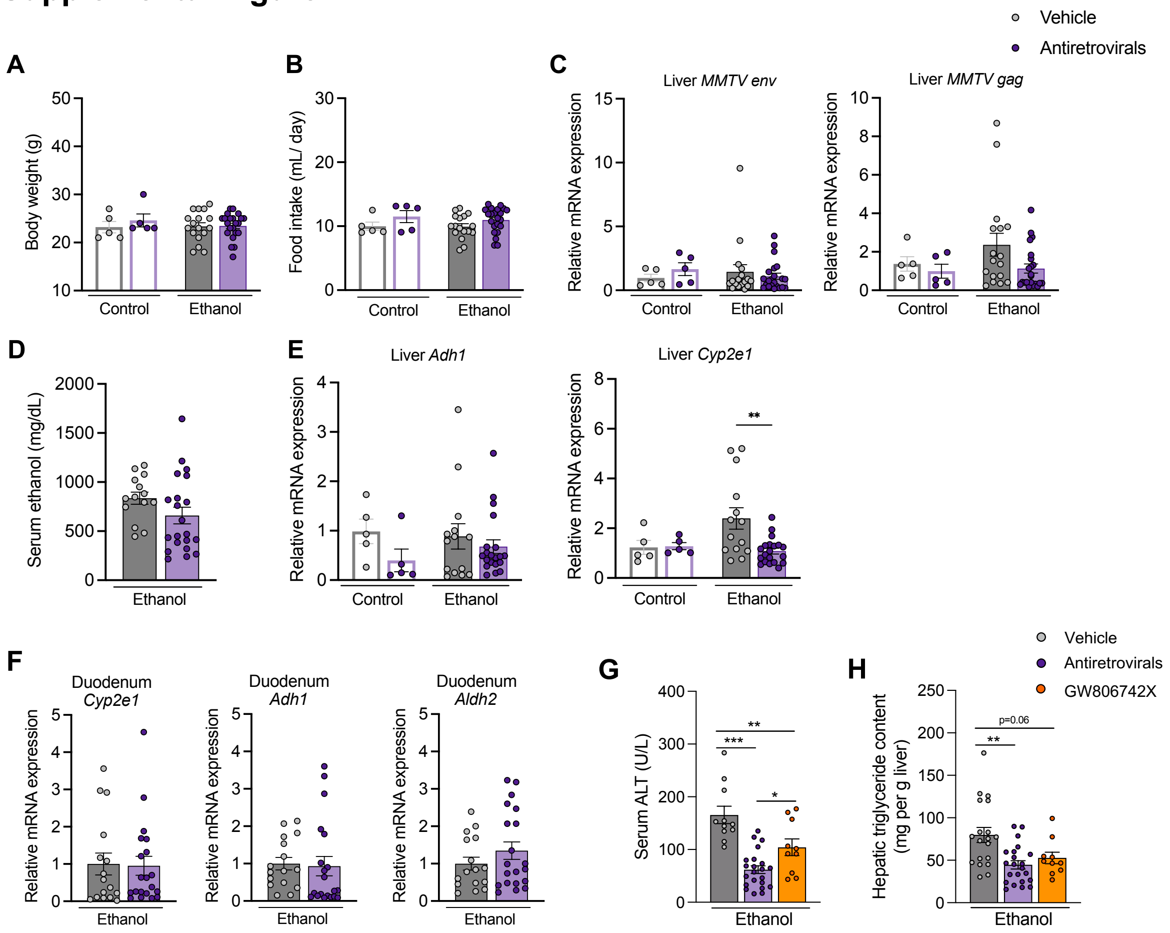

Supplemental Figure 4. Antiretroviral treatment in microbiota humanized mice subjected to chronic–binge ethanol feeding

(a–e) Germ-free C57BL/6 mice were colonized with stool from patients with alcohol-associated hepatitis (AH) and treated with a combination of antiretrovirals (emtricitabine 660 μM, tenofovir 314 μM, and nevirapine 375 μM) in drinking water after second colonization and then in liquid diet. Gnotobiotic mice were fed oral isocaloric (control) or chronic–binge ethanol diets. **(a)** Body weight. **(b)** Food intake of liquid diet. **(c)** Hepatic levels of mouse mammary tumor virus (MMTV) *env* and *gag* mRNA. **(d)** Serum levels of ethanol. **(e)** Hepatic levels of mRNAs encoding *Adh1* and *Cyp2e1*. **(f)** Duodenal levels of mRNAs encoding *Adh1, Aldh2* and *Cyp2e1*. **(g–h)** Germ-free C57BL/6 mice were colonized with stool from patients with alcohol-associated hepatitis (AH) and treated with a combination of antiretrovirals (emtricitabine 660 μM, tenofovir 314 μM, and nevirapine 375 μM) in drinking water after second colonization and then in liquid diet. A subset of mice was treated with the Mlkl inhibitor GW806742X at a dose of 2 mg/kg by intraperitoneal injections three times a week. Gnotobiotic mice were fed oral isocaloric (control) or chronic–binge ethanol diets. Note, mice treated with vehicle or antiretrovirals are the same as in Fig. 5g and h. **(g)** Serum levels of ALT. **(h)** Hepatic triglyceride content. Results were generated from two **(a–h)** technical replicates. P values among groups were determined by one-way analysis of variance (ANOVA) with Tukey's post hoc test **(a–c, e, g–h)** or Mann-Whitney U test **(d, f)**. Results are expressed as mean ± SEM. $^*P < 0.05$, $^{**}P < 0.01$, $^{***}P < 0.001$.

Supplemental Figure 5

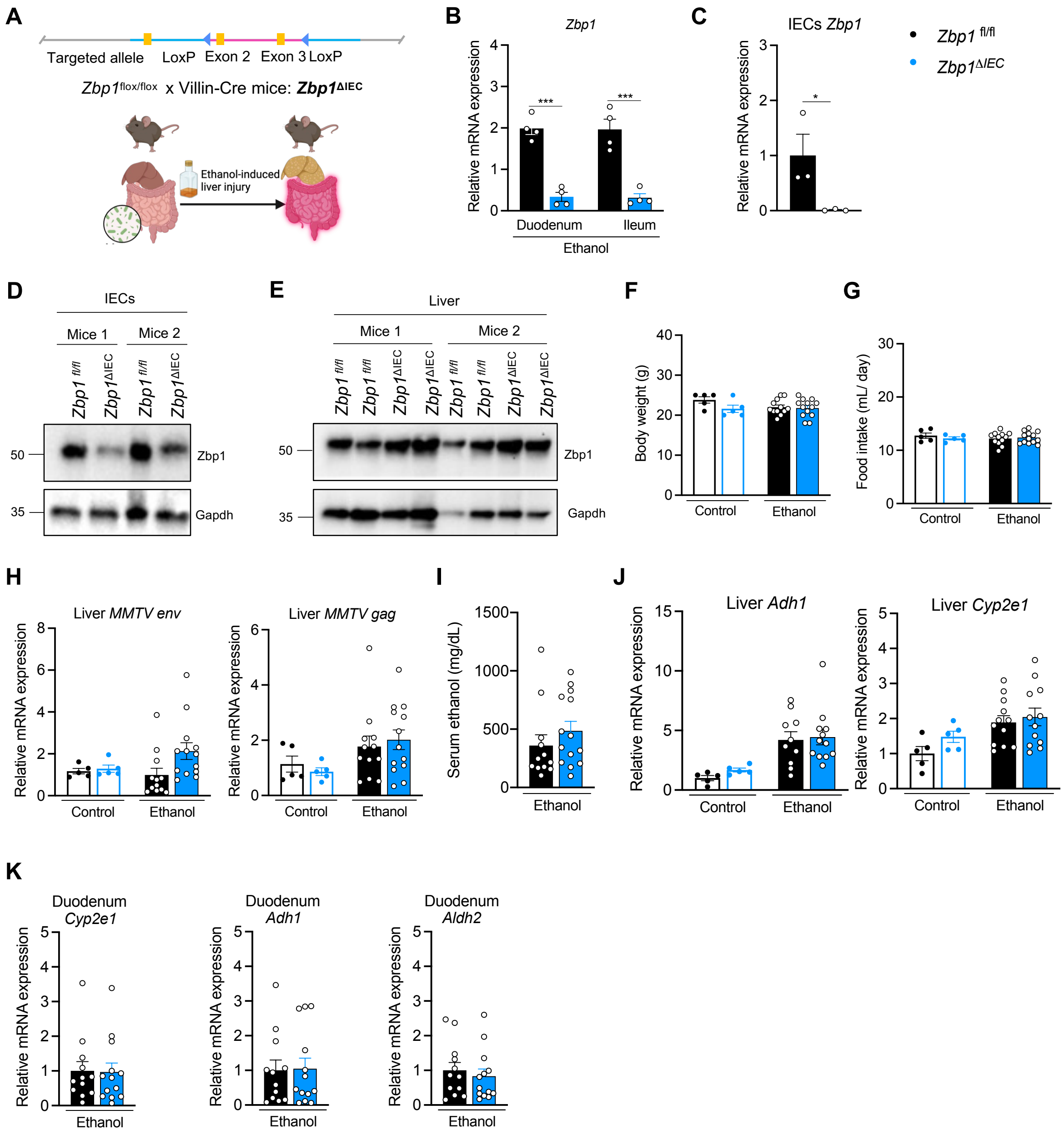

Supplemental Figure 5. Deficiency of *Zbp1* in intestinal epithelial cells and ethanol-induced liver disease

(a) A *Zbp1* conditional knockout mouse by CRISPR/Cas-mediated genome engineering was generated and crossed to villin-Cre transgenic mice to create mice with an intestinal epithelial cell (IEC)-specific deletion of *Zbp1* ($Zbp1^{\Delta IEC}$). $Zbp1^{\Delta IEC}$ and their littermate $Zbp1^{fl/fl}$ mice were fed oral isocaloric (control) or chronic–binge ethanol diets. **(b)** Levels of *Zbp1* mRNA in the duodenum and ileum. **(c)** Levels of *Zbp1* mRNA in intestinal epithelial cells isolated from the small intestine. **(d)** Intestinal epithelial cells were isolated from ethanol-fed mice and immunoblots were performed for Zbp1 and Gapdh. **(e)** Immunoblots of hepatic Zbp1 and Gapdh. **(f)** Body weight. **(g)** Food intake of liquid diet. **(h)** Hepatic levels of mouse mammary tumor virus (MMTV) *env* and *gag* mRNA. **(i)** Serum levels of ethanol. **(j)** Hepatic levels of mRNAs encoding *Adh1* and *Cyp2e1*. **(k)** Duodenal levels of mRNAs encoding *Adh1, Aldh2* and *Cyp2e1*. Results were generated from two technical replicates. P values among groups were determined by one-way analysis of variance (ANOVA) with Tukey's post hoc test **(b, f–h, j)** or Mann-Whitney U test **(c, i, k)**. Results are expressed as mean ± SEM.

Supplemental Figure 6

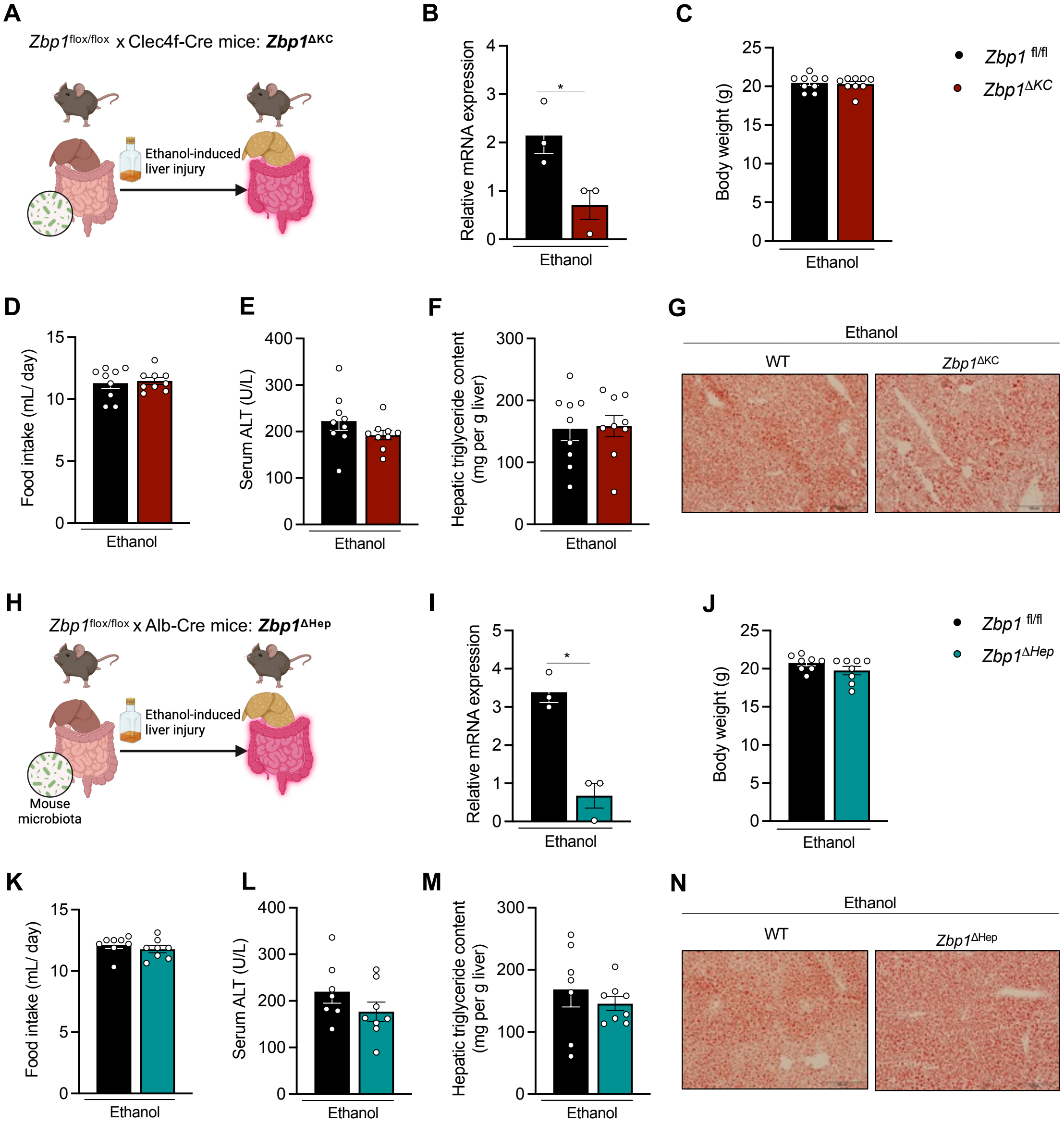

Supplemental Figure 6. Deficiency of *Zbp1* in Kupffer cells or hepatocytes does not protect from ethanol-induced liver disease

(a) *Zbp1*$^{fl/fl}$ mice were crossed to Clec4f-Cre transgenic mice to create mice with an Kupffer cell-specific deletion of *Zbp1* (*Zbp1*$^{\Delta KC}$). *Zbp1*$^{\Delta KC}$ and their littermate *Zbp1*$^{fl/fl}$ mice were fed oral isocaloric (control) or chronic–binge ethanol diets. **(b)** Levels of *Zbp1* mRNA in isolated Kupffer cells. **(c)** Body weight. **(d)** Food intake of liquid diet. **(e)** Serum levels of ALT. **(f)** Hepatic triglyceride content. **(g)** Representative images of liver sections stained with Oil Red O; scale bars, 100 μm. **(h)** *Zbp1*$^{fl/fl}$ mice were crossed to albumin-Cre transgenic mice to create mice with a hepatocyte-specific deletion of *Zbp1* (*Zbp1*$^{\Delta Hep}$). *Zbp1*$^{\Delta Hep}$ and their littermate *Zbp1*$^{fl/fl}$ mice were fed oral isocaloric (control) or chronic–binge ethanol diets. **(i)** Levels of *Zbp1* mRNA in isolated hepatocytes. **(j)** Body weight. **(k)** Food intake of liquid diet. **(l)** Serum levels of ALT. **(m)** Hepatic triglyceride content. **(n)** Representative images of liver sections stained with Oil Red O; scale bars, 100 μm. Results were generated from at least two technical replicates. P values among groups were determined by Mann-Whitney U test. Results are expressed as mean ± SEM.

Supplemental Figure 7

Uncropped blots Figure 2d

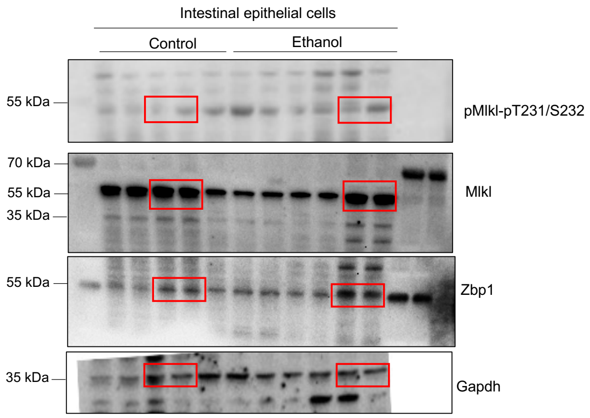

Uncropped blots Figure 2g

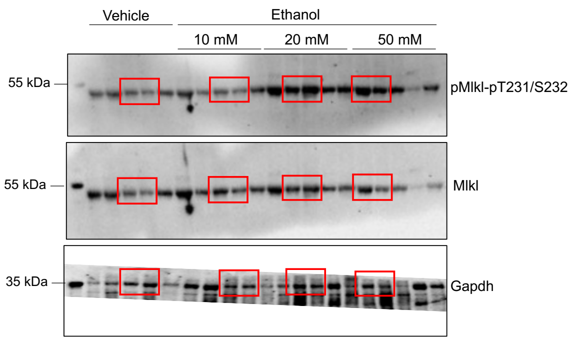

Uncropped blots Figure 2h

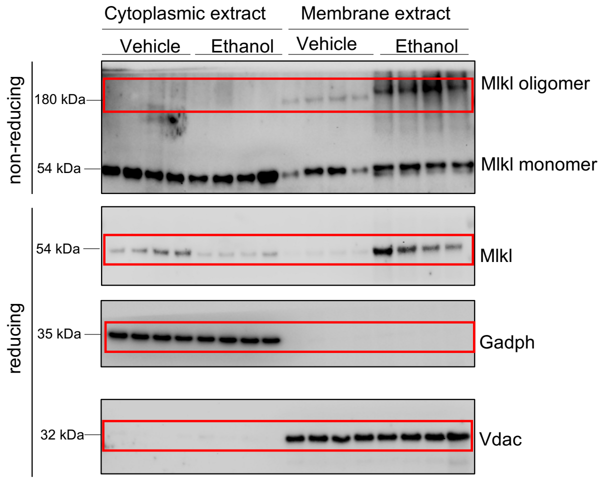

Uncropped blots Figure 3d

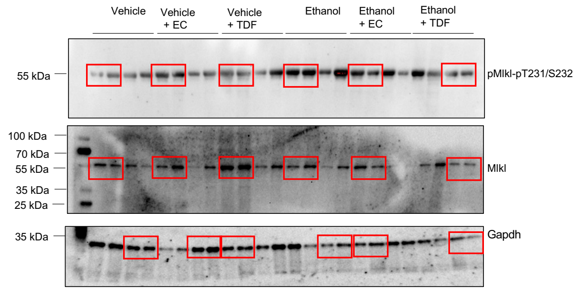

Uncropped blots Figure 3i

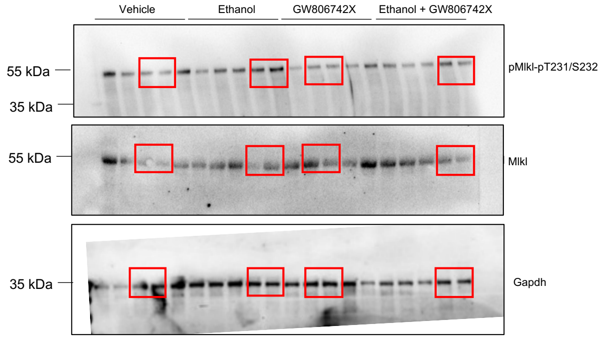

Uncropped blots Figure 5e

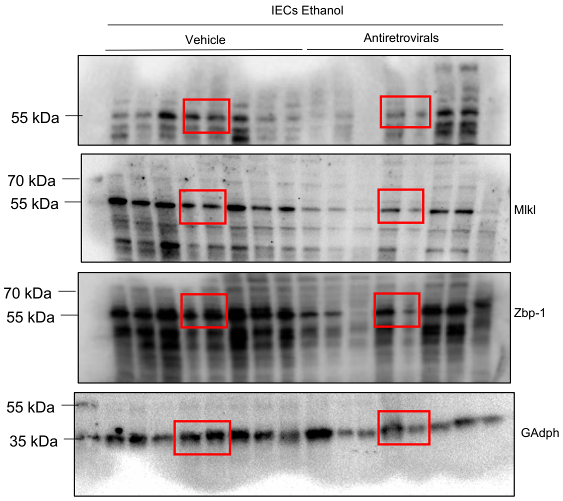

Supplemental Figure 7

Uncropped blots Figure 6h

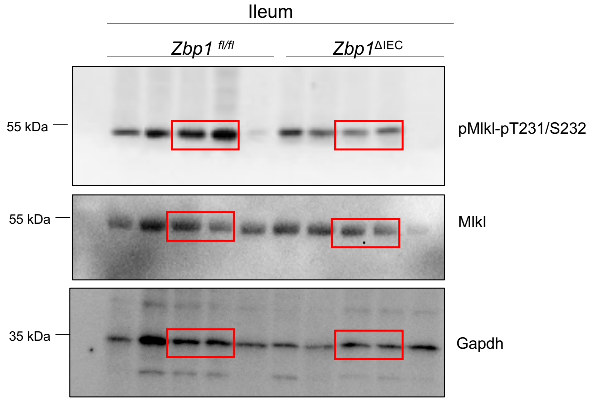

Uncropped blots Supplementary Figure 3b

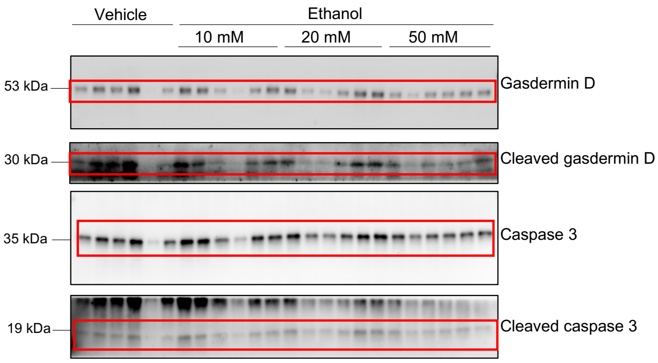

Uncropped blots Supplementary Figure 5d,e

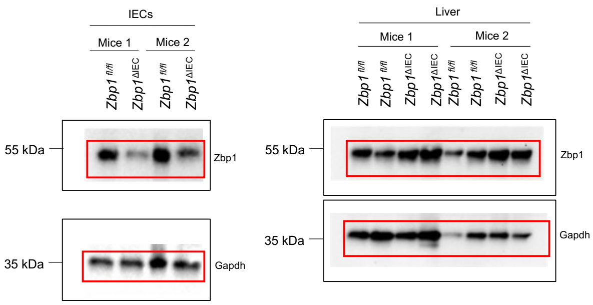

Supplemental Figure 7. Uncropped Blots

Supplementary Table 1: Baseline demographic and laboratory data of controls and patients with alcohol use disorder (duodenal biopsies)

	**Controls** (n=16)	**Alcohol use disorder** (n=44)
Gender (female), n=60	7 (44%)	14 (32%)
Age (years), n=60	46 [22;72]	51.0 [24.0;73.0]
BMI (kg/m^2), n=60	24.1 [19.6;31.8]	25.3 [16.2;36.6]
AST (IU/L), n=44		75.0 [15.0;436.0]
ALT (IU/L), n=44		44.0 [11.0;567.0]
GGT (IU/L), n=44		157.0 [10.0;2040.0]
ALP (IU/L), n=44		85.0 [37.0;243.0]
Bilirubin (mg/dL), n=44		0.40 [0.20;2.70]
Albumin (g/L), n=44		4.65 [0.1-5.6]
INR, n=43		1.0 [0.8;1.39]
Creatinine, n=44		0.7 [0.4;1.1]
CAP, n=44		297.0 [166.0;384.0]
Liver stiffness (kPa), n=44		7.0 [3.1,75.0]
Fibrosis stage, n=44		
F0/F1		26 (59%)
F2-F4		18 (41%)

Values are presented as median with range in brackets for continuous variables or number and percentage in brackets for categorical variables. Percentages are calculated

based on the actual number of patients in each group where the respective data was available. The number of subjects for which the respective data was available is indicated in the first column. ALP, alkaline phosphatase; ALT, alanine aminotransferase; AST, aspartate aminotransferase; BMI, body mass index; CAP, Controlled attenuation parameter; GGT, gamma-glutamyl transferase; INR, international normalized ratio.

Supplementary Table 2: Baseline demographic and laboratory data of controls and patients with alcohol use disorder (liver biopsies)

	**Controls** (n=5)	**Alcohol-Use Disorder** (n=27)
Gender (female), n=27	NA	5 (19%)
Age (years), n=27	NA	51.0 [23.0;73.0]
BMI (kg/m^2), n=27	NA	26.0 [20.2;33.8]
AST (IU/L), n=27		82.0 [17.0;348.0]
ALT (IU/L), n=27		51.0 [17.0;300.0]
GGT (IU/L), n=27		300.0 [16.0;2040.0]
ALP (IU/L), n=27		98.0 [49.0;243.0]
Bilirubin (mg/dL), n=27		0.70 [0.30;2.70]
Albumin (g/L), n=27		4.50 [3.20-5.60]
INR, n=24		1.07 [0.92;1.40]
Creatinine, n=27		0.76 [0.39;1.06]
CAP, n=27		332.0 [204.0;388.0]
Liver stiffness (kPa), n=27		12.4 [7.6;45.0]
Fibrosis stage, n=27, n (%)		
F0/F1		8 (30%)
F2-F4		19 (70%)

Values are presented as median with range in brackets for continuous variables or number and percentage in brackets for categorical variables. Percentages are calculated based on the actual number of patients in each group where the respective data was

available. The number of subjects for which the respective data was available is indicated in the first column. ALP, alkaline phosphatase; ALT, alanine aminotransferase; AST, aspartate aminotransferase; BMI, body mass index; CAP, Controlled attenuation parameter; GGT, gamma-glutamyl transferase; INR, international normalized ratio.

Supplementary Table 3: Primers used in this study.

Gene	Primer	Sequence
Mouse 18S	F	5'-AGTCCCTGCCCTTTGTACACA -3'
	R	5'-CGATCCGAGGGCCTCACTA -3'
Mouse Adh1	F	5'-GGGTTCTCAACTGGCTATGG-3'
	R	5'-ACAGACAGACCGACACCTCC-3'
Mouse Aldh2	F	5'-TTCCCACCGTCAACCCTTC-3'
	R	5'-CCAATCGGTACAACAGCCG-3'
Mouse Ccl2	F	5'-ATTGGGATCATCTTGCTGGT-3'
	R	5'-CCTGCTGTTCACAGTTGCC-3'
Mouse Cyp2e1	F	5'-GGGACATTCCTGTGTTCCAG-3'
	R	5'-CTTAGGGAAAACCTCCGCAC-3'
Mouse Cxcl1	F	5’-TGCACCCAAACCGAAGTC-3’
	R	5’-GTCAGAAGCCAGCGTTCACC-3’
Mouse Cxcl2	F	5’-AAAGTTTGCCTTGACCCTGAA-3’
	R	5’-CTCAGACAGCGAGGCACATC-3’
Mouse MMTV env [(1)]	F	5’-CGGAACGGACTCACCATAGG-3’
	R	5’-CAGATTGGTGATTCGGCATCT-3’
Mouse MMTV gag [(1)]	F	5’-GAAGAAACCTCAGGCACTCAGA-3’
	R	5’-GCCATCGCTCCTGCAAAA-3’
Mouse eMLV env [(2)]	F	5’-CCAGGGACCACCGACCCACCG-3’
	R	5’-TAGTCGGTCCCGGTAGGCCTCG-3’
Mouse pMLV/mpMLV [(2)]	common F	5’-CCGCCAGGTCCTCAATATAG-3’

Mouse pmLV [2]	R	5’-AGAAGGTGGGGCAGTCT-3’
Mouse mpMLV [2]	R	5’-CGTCCCAGGTTGATAGAGG-3’
E. Coli [3]	F	5’- CATGCCGCGTGTATGAAGAA-3’
	R	5’- CGGGTAACGTCAATGAGCAAA-3’
Mouse Zbp1	F	5’- GCTATGACGGACAGACGTGG-3’
	R	5’- TGTTGACCGGATTGTGCTGA-3’
Human HERV-K [4] (qPCR for lentiviral overexpression of HERV-K)	F	5′-TCTACCCTTGGGAATGGGGA-3′
	R	5′-AGCAGAATACGGTGTTGCCA-3′
Human HERV-K env [5]	F	5’-CACAACTAAAGAAGCTGACG-3’
	R	5’-CATAGGCCCAGTTGGTATAG-‘3
Human HERV-K pol [6]	F	5’-ATCCCAAAAGATTGGCCTTTA-3’
	R	5’-TTAAGCATTCCCTGAGGTAACA-3’
Human HERV-H env [7]	F	5’-TTCACTCCATCCTTGGCTAT-3’
	R	5’-CGTCGAGTATCTACGAGCAAT-3’
Human HERV-P env [5]	F	5’-CAAGATTGGGTCCCCTCAC-3’
	R	5’-CCTATGGGGTCTTTCCCTC-3’

References

1. Zhang G, Chen M, Graham D, Subsin B, McDougall C, Gilady S, et al. Mouse mammary tumor virus in anti-mitochondrial antibody producing mouse models. J Hepatol. 2011;55(4):876-84.
2. Lima-Junior DS, Krishnamurthy SR, Bouladoux N, Collins N, Han SJ, Chen EY, et al. Endogenous retroviruses promote homeostatic and inflammatory responses to the microbiota. Cell. 2021;184(14):3794-811 e19.
3. Arthur JC, Gharaibeh RZ, Muhlbauer M, Perez-Chanona E, Uronis JM, McCafferty J, et al. Microbial genomic analysis reveals the essential role of inflammation in bacteria-induced colorectal cancer. Nat Commun. 2014;5:4724.
4. Dembny P, Newman AG, Singh M, Hinz M, Szczepek M, Kruger C, et al. Human endogenous retrovirus HERV-K(HML-2) RNA causes neurodegeneration through Toll-like receptors. JCI Insight. 2020;5(7).
5. Dolci M, Favero C, Toumi W, Favi E, Tarantini L, Signorini L, et al. Human Endogenous Retroviruses Long Terminal Repeat Methylation, Transcription, and Protein Expression in Human Colon Cancer. Front Oncol. 2020;10:569015.
6. Manghera M, Ferguson J, Douville R. ERVK polyprotein processing and reverse transcriptase expression in human cell line models of neurological disease. Viruses. 2015;7(1):320-32.
7. Rhyu DW, Kang YJ, Ock MS, Eo JW, Choi YH, Kim WJ, et al. Expression of human endogenous retrovirus env genes in the blood of breast cancer patients. Int J Mol Sci. 2014;15(6):9173-83.
