# Supplementary material for: Activation of intestinal endogenous retroviruses by alcohol exacerbates liver disease
Source: J Clin Invest. 2025 May 13;135(13):e188541. doi: 10.1172/JCI188541 (PMC12208555; doi:10.1172/JCI188541)

Supplemental Figure 7

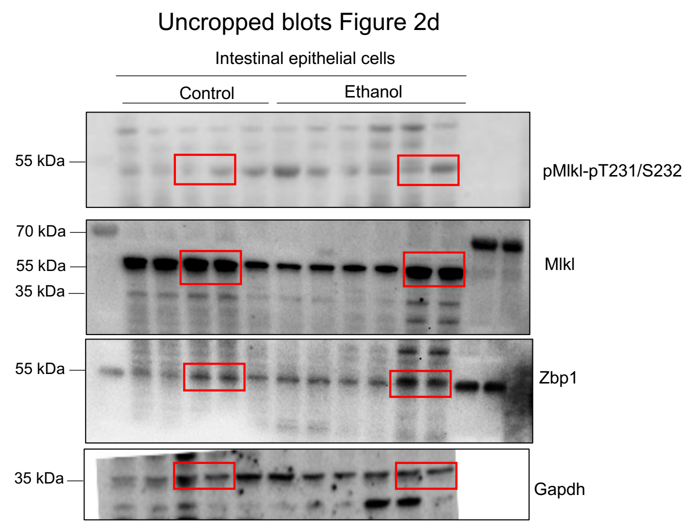

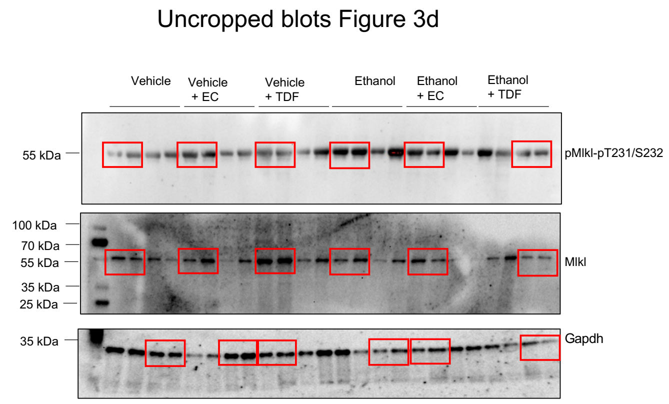

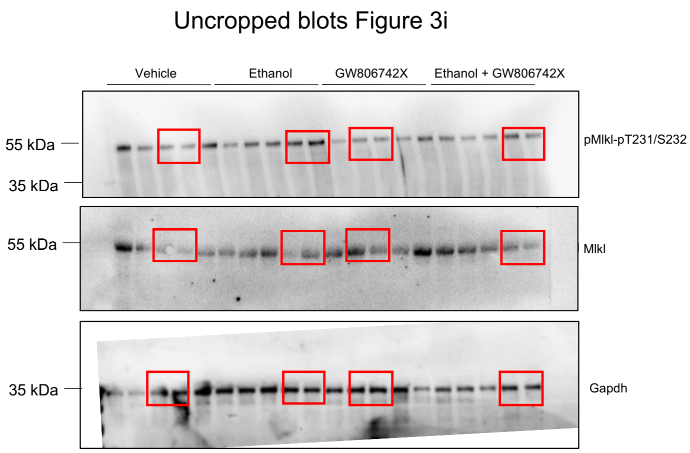

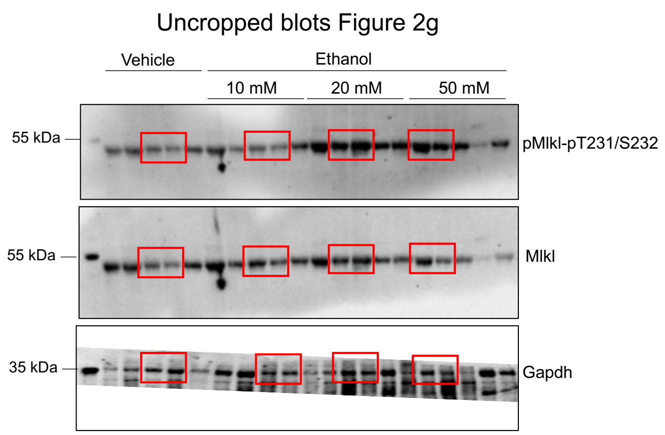

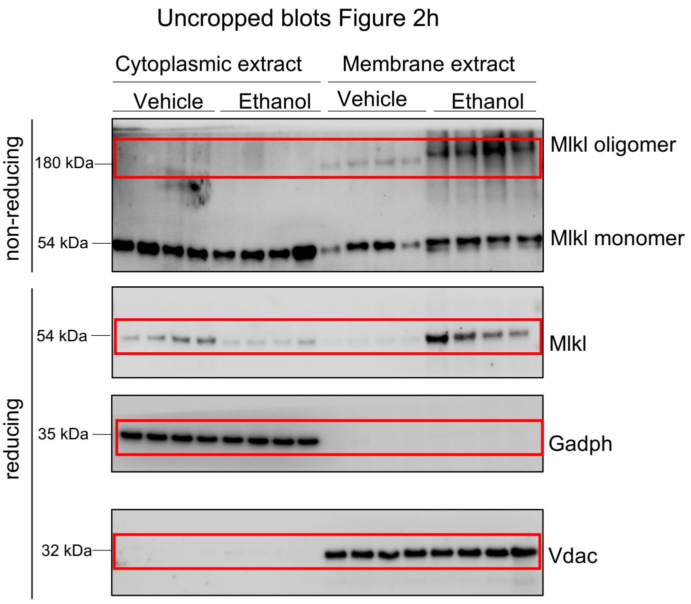

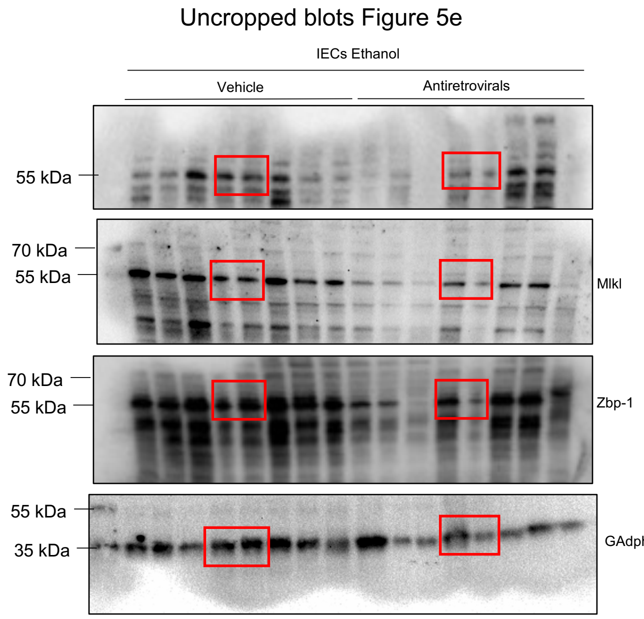

Supplemental Figure 7

Uncropped blots Figure 6h

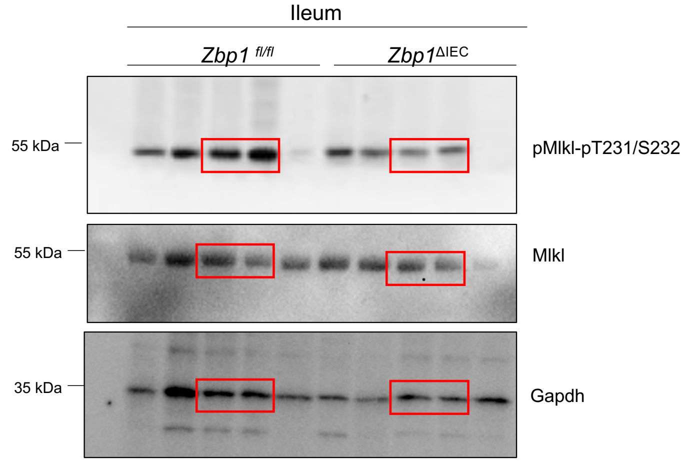

Uncropped blots Supplementary Figure 3b

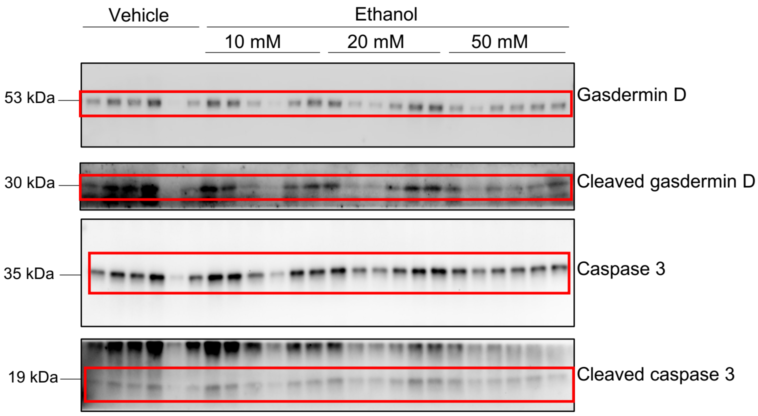

Uncropped blots Supplementary Figure 5d,e

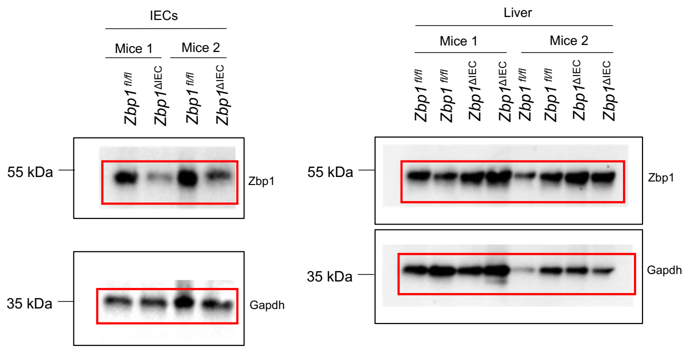

Supplement: Unedited blot and gel images [file jci-135-188541-s259.pdf]
